# Utilising public sequence databases to investigate genetic diversity of stoneflies in Medvednica Nature Park

**DOI:** 10.3897/BDJ.12.e121398

**Published:** 2024-04-18

**Authors:** Dora Kermek, Nikola Pischiutta, Dora Hlebec, Ignac Sivec, Mladen Kučinić

**Affiliations:** 1 University of Zagreb, Faculty of Science, Department of Biology, Zagreb, Croatia University of Zagreb, Faculty of Science, Department of Biology Zagreb Croatia; 2 Slovenian Museum of Natural History, Ljubljana, Slovenia Slovenian Museum of Natural History Ljubljana Slovenia

**Keywords:** Croatia, DNA barcoding, morphology, intraspecific haplotype variation, phylogeny, Plecoptera

## Abstract

In Medvednica Nature Park, near Croatia's capital Zagreb, urbanisation significantly impacts the fauna. Comprehensive field research has never been conducted in this area, despite the presence of diverse microhabitats and the discovery of several rare species previously unknown in the Croatian fauna. This study provides the Park with first insight into the genetic and morphological diversity of stoneflies, one of the most endangered groups of organisms. Phylogenetic reconstructions and species delineation methods revealed intraspecific haplotype variation in most species (e.g. *Brachypteraseticornis*, *Isoperlagrammatica* and *Leuctrabraueri*), except for *Leuctraprima*. Additionally, our study has identified isolated populations that merit further in-depth investigation concerning morphology, genetics and ecology.

## Introduction

More than 3700 species are described within the order Plecoptera, commonly known as stoneflies (DeWalt and Ower 2019). Species mostly prefer cold, fast-flowing streams with rocky bottoms and high oxygen levels. Only a few inhabit standing water or sandy substrates, such as *Nemouracinerea* (Retzius, 1783) and *Marthameavitripennis* (Burmeister, 1839). Together with caddisflies (Trichoptera) and mayflies (Ephemeroptera), stoneflies belong to the EPT (Ephemeroptera, Plecoptera, Trichoptera) group and are considered one of the most sensitive organisms to environmental pollution. This characteristic makes them excellent bioindicators of water quality (Fochetti and de Figueroa 2006).

Medvednica Nature Park is a mountainous region located near Zagreb, the capital and largest city of Croatia. More than 70 streams and 200 springs have been documented in the Park (Harmel et al. 2015). However, freshwater habitats are unfortunately facing threats from urbanisation and alterations in watercourses. These factors alter the appearance of these habitats (Harmel et al. 2015) and cause nutrient enrichment (Poulton et al. 2015), which boosts aquatic insect biomass. Nonetheless, they may also drive extinction in groups such as EPT, which are amongst the most endangered (Poulton et al. 2015). Despite the area's richness in various microhabitats and high biodiversity, which includes several rare species first discovered in the Croatian flora and fauna, such as the orchid *Epipogiumaphyllum* Swartz 1814 (Šegota and Alegro 2011) and the moth *Acontiacandefacta* Hubner, 1831 (Koren 2019), comprehensive field research on any group of organisms followed by the application of molecular methods has never been conducted in this region.

This study provides the first insight into the genetic and morphological diversity of stoneflies in the Medvednica Nature Park, thereby contributing to the faunal knowledge of the area. Additionally, it establishes phylogenetic and phylogeographical relationships between populations of widelydistributed species in Europe: *Isoperlagrammatica* (Poda, 1761), *Leuctrabraueri* (Kempny, 1898), *Leuctraprima* (Kempny, 1899) and *Brachypteraseticornis* (Klapálek, 1902). These relationships enhance understanding of diversification patterns and the biogeographic history of selected stonefly species, emphasising populations that warrant further detailed morphological, genetic and ecological investigation.

## Material and methods

### Sampling sites

**Figure 1.**
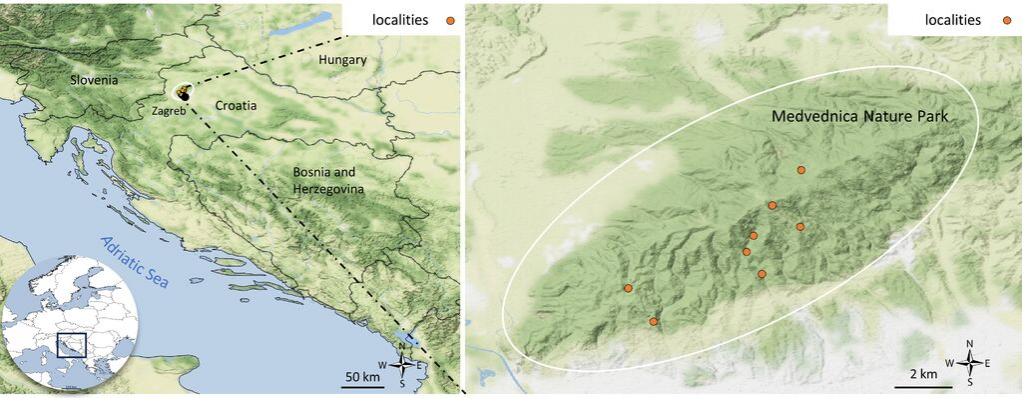
Sampling sites on the map of Medvednica Nature Park.

Specimens were collected at eight sampling sites in Medvednica Nature Park between June 2021 and March 2022 (Suppl. material 1, Fig. 1), with the permission of the Ministry of Economy and Sustainable Development of the Republic of Croatia (UP/I612-07/21-48/73). Adults were collected using an entomological net, while larvae were collected by hand under stones and branches. All collected individuals were preserved in 96% ethanol and stored at +4°C.

### Morphological examination

Morphological determination of adults was conducted using a ZEISS SteREO Discovery V20 stereomicroscope along with various identification keys (Illies 1955, Kaćanski and Zwick 1970, Sivec and Stark 2002, Zwick 2004, Murányi 2011). The characters used to distinguish species included the patterns of colouration of the head and pronotum, as well as the genital apparatus in both males and females. Larval identification was conducted through sequence comparison with entries available in databases.

### DNA extraction, amplification and sequencing

Total genomic DNA was isolated from the tissues of one to three legs of adults and larvae, depending on the specimen's size, using the GenElute Mammalian Genomic DNA Miniprep Kit (Sigma-Aldrich, Germany), following the manufacturer's instructions. To amplify the DNA barcode region, we utilised the universal primer set: LCO-1490 and HCO-2198 (Folmer et al. 1994). PCR reactions and subsequent purifications were carried out following the protocols outlined in Hlebec et al. (2022). Sequencing was performed by Macrogen Inc. (Amsterdam, Netherlands).

### Sequence editing

Chromatograms were analysed and edited in Geneious Prime 2022.1 (Biomatters, Auckland, New Zealand). Misread nucleotides were manually corrected. The presence of stop codons in all sequences was checked using Mesquite (Maddison and Maddison 2021). The BOLD Identification Engine (Ratnasingham and Hebert 2007) and NCBI BLAST search were used to confirm the authenticity of the amplified product.

### Phylogenetic and phylogeographic analyses

For phylogenetic and phylogeographic analyses, we selected species with a broader distribution and a larger number of *COI* sequences available in the BOLD and GenBank databases. The selected species were *Brachypteraseticornis*, *Isoperlagrammatica*, *Leuctrabraueri* and *Leuctraprima*. Downloaded sequences were aligned using the ClustalW multiple alignment tool (Larkin et al. 2007) and poor-quality sequences, sequences shorter than 500 bp or those containing stop codons, were excluded from further analyses. Subsequently, sequences were collapsed into unique haplotypes using the online service FaBox v.1.61 (Villesen 2007). *Perlapallida* Guérin-Méneville, 1838 (CROPL090-19), *Isoperlagrammatica* (CROPL154-21) and *Nemouraavicularis* Morton, 1894 (CROPL015-21) were used as outgroups. Phylogenetic analyses were performed using the Maximum Likelihood (ML) approach in RaxML-HPC v.8 on XSEDE (Stamatakis 2014) through the CIPRES online service (Miller et al. 2010). Rapid bootstrap analysis was performed using the GTRGAMMA model with 1000 iterations. Nodes that received bootstrap values (RBS) higher than 70% were considered as supported. The resulting phylogenetic trees were visualised and edited using FigTree v.1.4.4. (Rambaut 2018) and iTOL (Letunic and Bork 2021). To investigate concordance between morphological and molecular species clustering, we applied different species delimitation methods: ASAP (Puillandre et al. 2020) using both *p*-distance and Kimura-2-Parameter (K2P) distance options, ABGD (Puillandre et al. 2011), bPTP (Zhang et al. 2013) and the BIN (Barcode Index Number) system. Additionally, the ranges of intraspecific uncorrected pairwise distances (*p*-distances, hereafter referred to as "genetic distance") were calculated for each selected species using the programme MEGA 11 (Tamura et al. 2021). TCS phylogenetic networks (Clement et al. 2002) were created using the statistical parsimony method in the PopART programme (Leigh and Bryant 2015). Geographical distributions of haplotypes were made using the Cartopy package v.0.19 in Python v.3.8.

## Data resources

The collected material is deposited in the Collection of Plecoptera Sivec & Hlebec (CPSH), curated at the Croatian Natural History Museum (CNHM) and is accessible through GBIF under the DOI:  https://doi.org/10.15468/7rydnq. All sequences are publicly accessible in the BOLD database under the DOI: https://doi.org/10.5883/DS-CROPLM, Zenodo database under the DOI: http://doi.org/10.5281/zenodo.10665368 and GenBank under the accession numbers PP214131–PP214155.

## Results

During field research, 101 stonefly individuals (76 adults and 25 larvae) were collected and 25 of them were selected for DNA extraction, spanning the morphological variability. A total of 14 species, six genera and five families were identified (Suppl. material 1). *Leuctrasignifera* Kempny, 1899 and *Leuctranigra* Olivier, 1811 were identified, based solely on morphology, as PCR reactions were unsuccessful. *Protonemurapraecox* Morton, 1894, *Isoperlagrammatica*, *Leuctraprima* and *Perlapallida* were identified through sequence comparison with entries available in databases. These species could not be distinguished, based on morphology due to deviations from the species descriptions.

Amongst the collected individuals, the genus *Leuctra* predominated with 71 specimens, of which 63 were identified as *Leuctrabraueri*, accounting for 70.3% and 62.4% of the total identified specimens, respectively. Remarkably, the discovery of *Leuctrabraueri* marks the first record of this species in the fauna of Croatia. The substantial number of adults collected suggests that the population on Medvednica Mountain is likely the largest in Croatia, as otherwise, the species would have been recorded earlier.

Furthermore, one adult *Leuctra* individual (CROPL384-22) could not be identified through morphology alone, because there is no comparative material available in the collections of the Croatian Natural History Museum and the Slovenian Museum of Natural History. This individual exhibited morphological similarities with the species *Leuctrasignifera*, *Leuctradalmoni* Vincon & Murányi, 2007, *Leuctracarpathica* Kis, 1966 and *Leuctraprima*.

The generated sequences were classified into eight existing BINs and four unique BINs (AES3404, AES9615, AES9453, AER8749) (Suppl. material 1). Species *Brachypteraseticornis*, *Isoperlagrammatica*, *Leuctrabraueri* and *Leuctraprima*, all recorded in Medvednica Nature Park, were selected for further phylogeographic analyses. Detailed results of the species delineation methods (ASAP and ABGD) are provided in Suppl. material 1. Further explanations for each analysed species can be found in their respective results sections.

### Brachypteraseticornis

**Figure 2.**
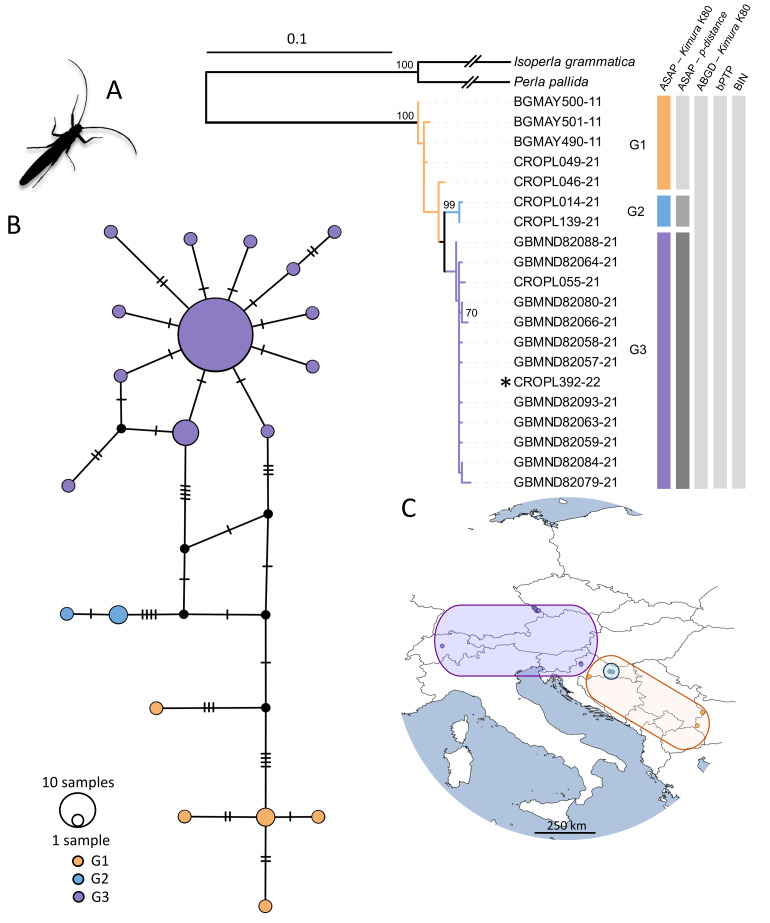
Phylogeographic analysis of *Brachypteraseticornis*, based on the *COI* gene fragment, 658 bp in length. **A** ML phylogenetic tree. The terminals correspond to the BOLD IDs. Individual collected in the present study is marked with an asterisk. Bars in different colours represent the results of species delimitation methods; **B** Phylogenetic network obtained by the statistical parsimony (TCS network). Colour coding corresponds to the groups in the species tree; **C** Geographical distribution of analysed haplotypes. Colour coding corresponds to the groups in the species tree and the TCS network.

The ASAP analysis, using both *p*-distance and K2P distance options, grouped haplotypes into three distinct groups. Conversely, the ABGD analysis, utilising the K2P distance option, grouped all haplotypes into a single group, as did the bPTP analysis. Notably, all analysed sequences were assigned to the same BIN (AAY5851) (Fig. 2). Specimen CROPL392-22 from this study was nested within Croatian specimens. The three groups identified by ASAP were also evident in the phylogenetic network (Fig. 2). Samples from Zrinska gora Mountain grouped with those from south-eastern Europe, Bulgaria and Serbia, while Medvednica Mountain samples grouped with those from central and western Europe (G3). The intraspecific genetic distances for *Brachypteraseticornis* ranged from 0.15% to 2.28% (Suppl. material 1).

### Leuctrabraueri

**Figure 3.**
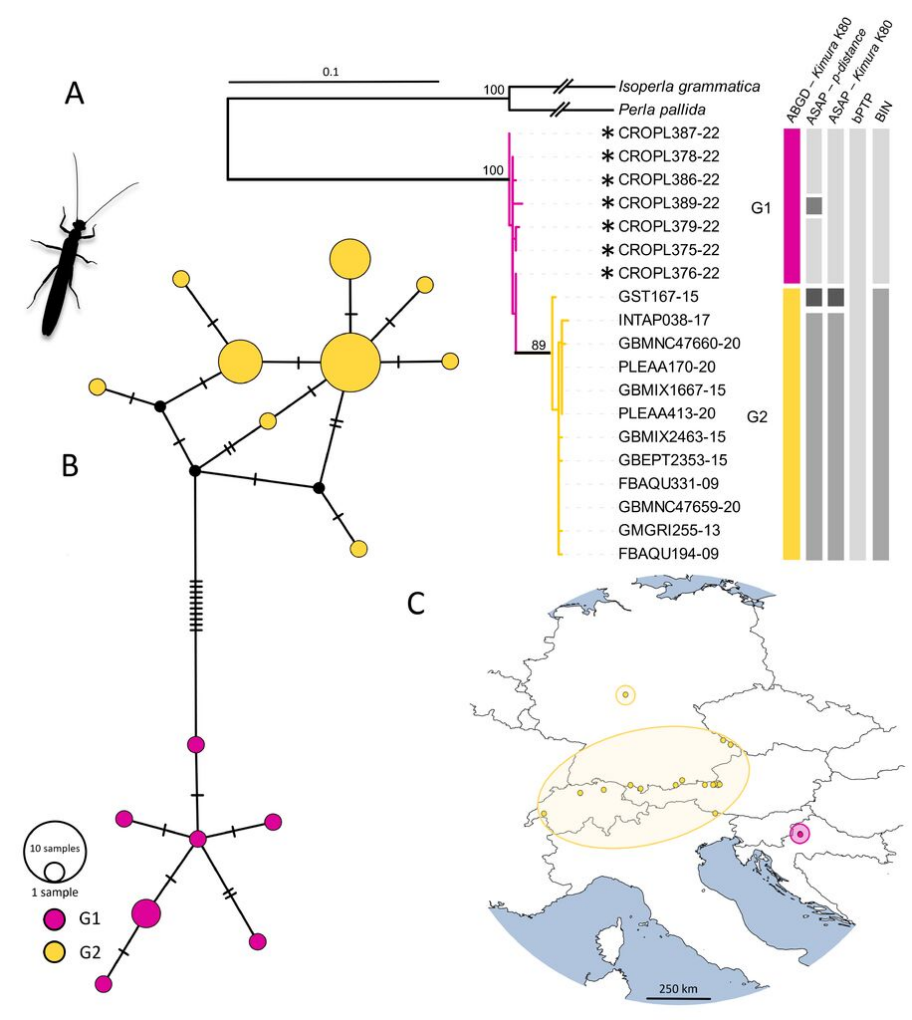
Phylogeographic analysis of *Leuctrabraueri*, based on the *COI* gene fragment, 658 bp in length. **A** ML phylogenetic tree. The terminals correspond to the BOLD IDs. Individuals collected in present study are marked with asterisks. Bars in different colours represent the results of species delimitation methods; **B** Phylogenetic network obtained by the statistical parsimony (TCS network). Colour coding corresponds to the groups in the species tree; **C** Geographical distribution of analysed haplotypes. Colour coding corresponds to the groups in the species tree and the TCS network.

The ASAP analysis, using the *p*-distance option, grouped haplotypes into four distinct groups (with lineage CROPL389-22 nested within G1). In contrast, the K2P distance option yielded three groups. ABGD analysis, also using the K2P distance option, grouped haplotypes into two groups, while bPTP analysis grouped all haplotypes into a single group. Sequences were assigned to two BINs (AAJ2415, AES9453), with specimens from this study belonging to a unique BIN. The species tree showed two clades, one nested within the other. Two groups identified by ABGD analysis were evident in the phylogenetic network (Fig. 3). Samples from Medvednica Mountain formed a distinct group (G1), while another group (G2) included samples from central Europe (Germany, Austria, Italy and Switzerland) (Fig. 3). The intraspecific genetic distances for *Leuctrabraueri* ranged from 0% to 2.46% (Suppl. material 1).

### Leuctraprima and Leuctra sp. ZB

Morphological examination of the *Leuctra* specimen (CROPL384-22) from Medvednica Mountain was inconclusive. It showed similarities to several species not represented in the BOLD and GenBank databases: *Leuctraprima*, *Leuctrasignifera*, *Leuctradalmoni* and *Leuctracarpathica*. Therefore, more detailed phylogenetic analyses, including sequences of those closely-related species, were omitted. *COI* sequence of collected specimen (CROPL384-22) showed the highest similarity to an undescribed *Leuctra* species (labelled as *Leuctra* sp. ZB, from Žumberačko gorje Mountain in Croatia, CROPL248-21) (Hlebec et al. 2022). Hence, we adopted the same designation here. Both of these lineages exhibited the closest morphological similarity to *Leuctraprima*.

**Figure 4.**
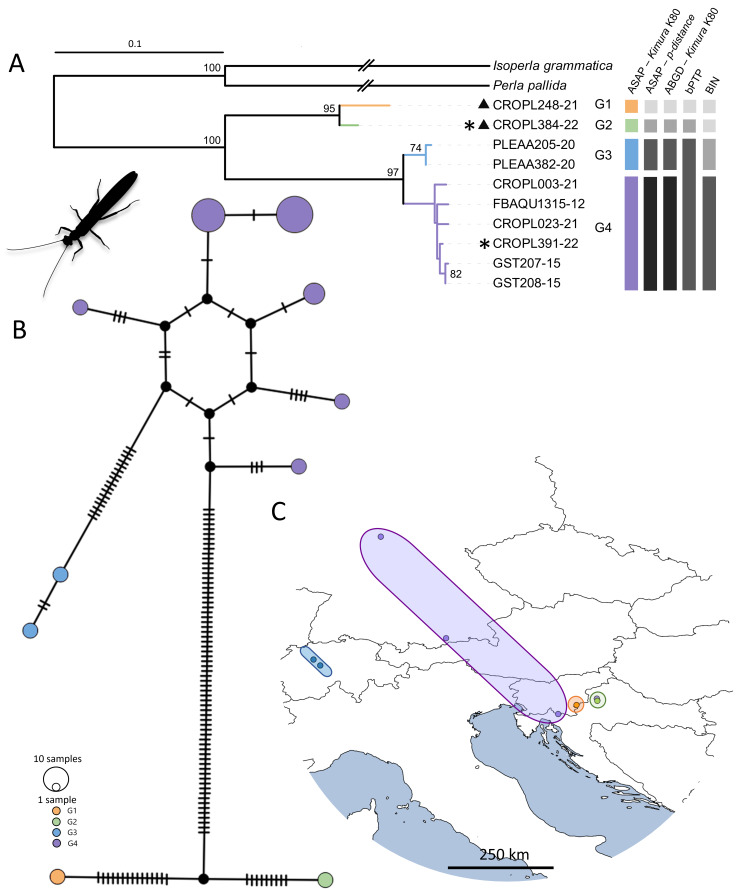
Phylogeographic analysis of *Leuctraprima* and *Leuctra* sp. ZB, based on the *COI* gene fragment, 658 bp in length. **A** – ML phylogenetic tree. The terminals correspond to the BOLD IDs. Individuals collected in present study are marked with an asterisk and *Leuctra* sp. ZB specimens with triangles. Bars in different colours represent the results of species delimitation methods. **B** – Phylogenetic network obtained by the statistical parsimony (TCS network). Colour coding corresponds to the groups in the species tree. **C** – Geographical distribution of analysed haplotypes. Colour coding corresponds to the groups in the species tree and the TCS network.

The species tree was characterised by two singletons and two highly-supported clades labelled as G1–G4 (Fig. 4), corresponding to two morphotypes: *Leuctra* sp. ZB and *Leuctraprima*. All species delimitation methods were congruent in delimiting four groups, except for bPTP, which clustered all *Leuctraprima* sequences and separated *Leuctra* sp. ZB (CROPL384-22 and CROPL248-21) into two distinct groups. Additionally, a unique BIN (AES3404) was assigned to specimen labelled *Leuctra* sp. ZB. (CROPL384-22) collected in this study.

The four groups identified by ASAP, ABGD and BIN assignment were confirmed in the phylogenetic network (Fig. 4). Interspecific genetic distances between *Leuctraprima* (G3 and G4) and *Leuctra* sp. ZB (G1 and G2) ranged from 12.0 to 13.5%, while intraspecific genetic distances for *Leuctraprima* (G3 and G4) and *Leuctra* sp. ZB (G1 and G2) reached a maximum of 3.65% (Suppl. material 1).

### Isoperlagrammatica

**Figure 5.**
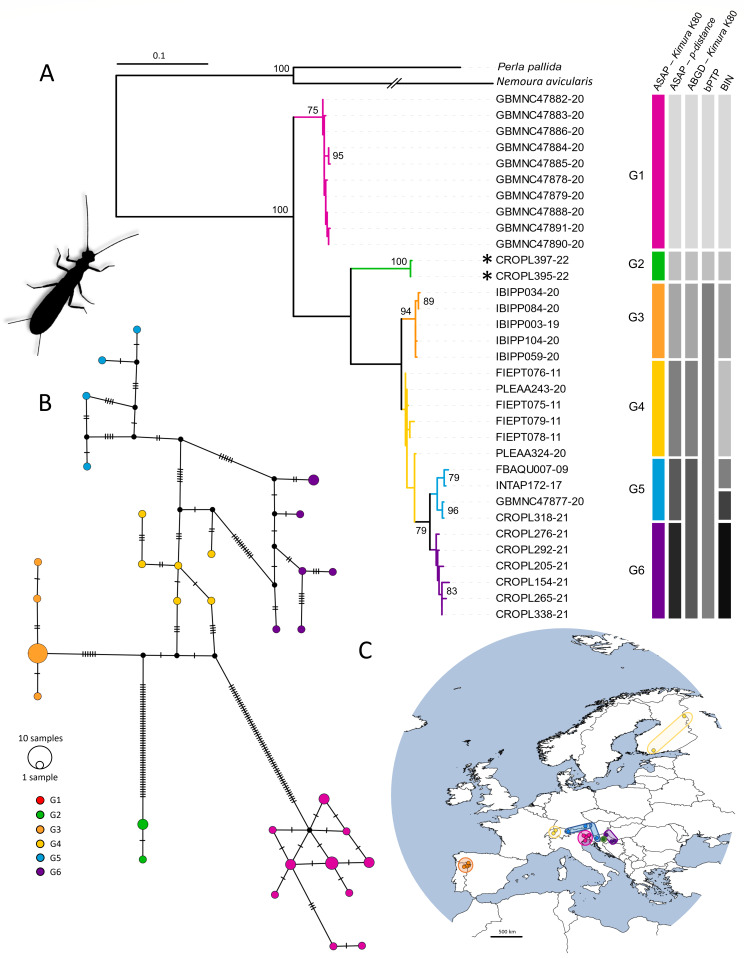
Phylogeographic analysis of *Isoperlagrammatica*, based on the *COI* gene fragment, 658 bp in length. **A** – ML phylogenetic tree. The terminals correspond to the BOLD IDs. Individuals collected in present study are marked with asterisks. Bars in different colours represent the results of species delimitation methods. **B** – Phylogenetic network obtained by the statistical parsimony (TCS network). Colour coding corresponds to the groups in the species tree. **C** – Geographical distribution of analysed haplotypes. Colour coding corresponds to the groups in the species tree and the TCS network.

ASAP analysis, using both the *p*-distance and K2P distance options, grouped haplotypes into six groups. ABGD analysis, using the K2P distance option, resulted in five groups, while bPTP analysis grouped haplotypes into three groups. Seven BINs were assigned: AAY9655, AEH6396, AEG4373, AEC9627, ACJ0709, AAK4351 and AER8749, with all specimens from this study falling into one distinct BIN. The species tree was characterised by four clades labelled as G1–G3 and G5–G6 (Fig. 5) and group G4 which indicates several singletons. The six groups identified by the ASAP analysis were also evident in the phylogenetic network (Fig. 5). Intraspecific genetic distances for *Isoperlagrammatica* ranged up to 11.7% (Suppl. material 1).

## Discussion

Recent studies (Hlebec et al. 2022) mark the beginning of comprehensive stonefly research in Croatia, expanding on previous localised investigations (Popijač and Sivec 2009, Popijač and Sivec 2010, Popijač and Sivec 2011). Through an integrative approach, we identified 14 stonefly species in the Medvednica Nature Park (Suppl. material 1), thereby enhancing species biomonitoring and enabling assessment of urbanisation impacts on fauna composition.

To explore genetic differentiation amongst widely-distributed stonefly species in Europe, we sequenced the *COI* gene fragment, a reliable gene fragment for determining inter- and intraspecific relationships (Hebert et al. 2003b). Based on phylogenetic reconstruction and the application of three distance-based delimitation approaches (ASAP, ABGD and BIN assignments), as well as one tree-based delimitation approach (bPTP), we determined intraspecific haplotype variation in *Brachypteraseticornis*, *Isoperlagrammatica* and *Leuctrabraueri*, with no such variation observed in *Leuctraprima*.

### Genetic homogeneity and dispersal of the species Bracyhpteraseticornis

*Brachypteraseticornis*, typically absent in Italy, inhabits southern and central Europe, favouring cold streams at altitudes from 300 to 2900 m, including the Alpine Region (Schmidt-Kloiber and Hering 2019).

Species delineation methods revealed up to three groups within *B.seticornis*, with intraspecific genetic distance up to 2.43%. The haplotypes were separated by a low number of mutations (Fig. 2). One specimen from Medvednica Mt. (CROPL392-22) aligns with samples from Germany and Switzerland (G3), while others from Croatia cluster with those from Bulgaria and Serbia (G1). The population (G2) between central (G3) and south-eastern Europe (G1) comprises the remaining Croatian individuals from Medvednica Mt. and Zrinska gora Mt. Based on the results of the phylogeographic analyses, we assume that the dispersal of the species was from southeast to central Europe (Fig. 2).

### Genus Leuctra – diversity and dispersal patterns

The genus *Leuctra* Stephens, 1836 is a dominant member of the Leuctridae family, characterised by small, dark-coloured species. Resolving phylogenetic relationships within *Leuctra* remains challenging, with overlapping intraspecific and interspecific genetic distances (Vitecek et al. 2017). To enhance our understanding, we analysed three species from the region: *Leuctrabraueri*, *Leuctraprima* and an undescribed species, labelled as *Leuctra* sp. ZB.

Both *L.braueri* and *L.prima* are found primarily in mountainous regions of central Europe, particularly the Carpathians, at altitudes above 200 m. *L.braueri* extends into the Dinaric Western Balkans, while *L.prima* inhabits the entire Balkans. These species favour cold streams (6 – 10°C) with neutral pH and either moderate to high (*L.prima*) or low (*L.braueri*) flow (Schmidt-Kloiber and Hering 2019).

Our discovery of *Leuctrabraueri* marks its first recorded presence in Croatia. It shows genetic divergence, with central European samples (G2) distinct from Croatian specimens (G1). Fewer mutational steps and low intraspecific genetic distances (1.84–2.46%) could suggest a relatively recent intraspecific split. The phylogenetic network hints at Croatian samples potentially being the diversification centre of the central-European lineage, suggesting a migration route from east or south Europe to the west. Furthermore, the question is, would that intraspecific structuring exist if samples from Slovenia and Austria had been included in the analysis? This question emphasises once again the consequences of undersampling in barcoding and phylogeographic studies.

We confirmed the genetic distinctiveness of the undescribed species, *Leuctra* sp. ZB, from its closest relative, *L.prima* (interspecific genetic distances were 12.02–13.55%). This exceeds the average interspecific genetic distances in Plecoptera, which is 11.56% (Zhou et al. 2010). All species delimitation methods separated two *Leuctra* sp. ZB samples (one from Medvednica Mt. and one from Žumberačko gorje Mt.) into distinct groups. In addition to the morphological similarities with *L.prima*, the specimen from Medvednica Mt. showed a resemblance to *L.dalmoni*, *L.carpathica* and *L.signifera*, which are distributed in central Europe and the Alps (Vinçon and Múranyi 2007). However, their emergence periods do not align with our specimen's late summer collection, as *L.carpathica* and *L.signifera* emerge during autumn and *L.dalmoni* emerges during winter and spring. Furthermore, the distribution ranges of *L.carpathica* and *L.signifera* exclude the Balkans and the Dinaric Region, as they are only recorded in the Alps (*L.carpathica* and *L.signifera*) and the Carpathians (*L.carpathica*) (Schmidt-Kloiber and Hering 2019). Regarding the other DNA barcoded *Leuctra* sp. ZB specimen from Žumberačko gorje Mountain, while the altitude range matches (between 200 and 800 m), the emergence periods do not, as this specimen was found during autumn. Although a longer emergence period is possible, the significant genetic differences between the samples should be considered. Collected specimens likely represent one or two endemic species, with the type localities in north-western Croatia. Detailed morphological examination, including analysis of additional genetic markers, will be necessary to distinguish and describe these species and determine their phylogenetic relationships. The challenge in determining relatedness within the genus *Leuctra* lies in the lack of genetic data for described species (e.g. *Leuctradalmoni*, *Leuctracarpathica* and *Leuctrasignifera*). It is regrettable that even the most recent study (Reding et al. 2023) did not incorporate molecular data in the description of *Leuctrapapukensis* Reding, Vinçon & Graf, 2023.

### Cryptic diversity of the species Isoperlagrammatica

*Isoperlagrammatica* is widely distributed across Europe, inhabiting altitudes from lowland to subalpine regions (0–2400 m) with no specific preference for water temperature or pH conditions (Schmidt-Kloiber and Hering 2019).

Phylogenetic reconstruction of *Isoperlagrammatica* revealed complex structuring. Delimited groups had distinct geographical distributions: Croatia (G6, G2), Switzerland (G4), Italy (G1), Austria (G3) and a group (G5) that includes samples from the Alpine Region and central Europe (Slovenia, Italy, Austria and Germany). Croatian individuals formed two separate lineages corresponding to different altitudes: a mountain lineage in the Medvednica Nature Park (G2, altitudes between 300 and 1000 m) and a lowland lineage (G6, altitudes below 200 m). Intraspecific genetic distances ranged from 9.02–11.7%, indicating significant genetic diversity. Expanding sampling to broader scales may increase intraspecific variation, while interspecific divergence impact is likely to decrease due to allopatric speciation dominance (Bergsten et al. 2012). Alpine Region samples were genetically closer to the lowland-Croatian lineage than to Medvednica Nature Park samples, suggesting exceptional isolation of the latter. Genetic distances between Medvednica Mt. population and others ranged from 7.93 to 10.4%, exceeding the maximum intraspecific genetic distances within the genus *Isoperla* (7.82%) (Hlebec et al. 2021), possibly indicating an undescribed species.

The widespread distribution of *I.grammatica* in Europe, along with its complex genetic differentiation, suggests the presence of cryptic diversity. Cryptic species lack morphological differences, but are often genetically distinct, forming highly-supported monophyletic lineages (Bickford et al. 2007). Following phylogenetic analyses, individuals are often re-examined to identify distinguishing morphological traits (Pauls et al. 2010). To gain a clearer understanding of the phylogenetic relationships and the delimitation of potential cryptic species within the *I.grammatica* species complex, a detailed morphological examination, including different life stages and eggs, supported by a multi-gene approach is required. The very recent genomic sequencing of a specimen from the River Test, Great Bridge, Hampshire (McSwan et al. 2023) will be used as a reference for future phylogenetic studies using new-generation techniques.

### Phylogenetic uniqueness of the samples collected in Nature Park Medvednica

Stoneflies, known for their poor flying abilities (Stewart 2009), tend to remain in specific areas, resulting in high rates of endemism, mutation accumulation and allopatric speciation. Phylogenetic and phylogeographic analyses of selected species recorded in the studied area (*Brachypteraseticornis*, *Isoperlagrammatica*, *Leuctrabraueri*) revealed high genetic diversity, except for *Leuctraprima*.

Phylogenetic reconstructions suggest that central European specimens of *Brachypteraseticornis* likely originated from eastern Europe, with a population on Medvednica Mt. bridging central and southeast Europe. A similar phylogenetic tree topology was observed in *Leuctrabraueri*, with specimens from Medvednica Mt. representing the first branch-off in species tree.

Individuals of *Leuctrabraueri*, *Isoperlagrammatica* and, potentially, the new species *Leuctra* sp. ZB on Medvednica Mt. exhibited genetic distinctiveness from other samples in central Europe (Germany, Austria, Switzerland and Italy).

### DNA barcoding in biodiversity research and conservation biology

DNA barcoding serves as a primary tool for specimen identification and biodiversity quantification. It is increasingly utilised for the discovery and description of new and cryptic species, as well as for associating immature and mature life stages (Hebert et al. 2003a, Hebert et al. 2003b, Hebert et al. 2004). The morphological analysis in this research aligns with DNA barcoding results, confirming its effectiveness (Hebert et al. 2004, Pauls et al. 2010). The comprehensiveness of the database for target group in the researched area is crucial for linking morphologically identified individuals with DNA barcodes. Hlebec et al. (2022) published a DNA barcoding reference library for stoneflies in Croatia, facilitating precise stonefly species identification.

Our study emphasises the importance of faunistic and phylogenetic analyses for identifying populations with unique genetic traits. This underscores the necessity for increased habitat protection and conservation efforts. DNA barcoding emerges as a crucial tool in conservation biology, aiding in the development of management plans for specific animal groups and areas.

Despite its utility, DNA barcoding encounters challenges. Species delimitation may not always be straightforward, especially with variations in intraspecific genetic diversity. Some species exhibit greater intraspecific genetic diversity, as seen in *Isoperlagrammatica*, while others show much lower intraspecific genetic diversity, such as *Bracyhpteraseticornis*. In unexplored areas or with lesser-known organisms, the method's efficacy can suffer due to database limitations. Taxonomic experts and detailed morphological analyses are crucial in such cases. BOLD and GenBank databases are valuable resources for European stonefly fauna research, particularly in well-sequenced regions like central and northern Europe, including Medvednica Nature Park.

## Conclusions

In our study, the DNA barcoding method demonstrated its efficiency and reliability in identifying samples collected in the Medvednica Nature Park. Furthermore, populations of the species *Brachypteraseticornis*, *Isoperlagrammatica* and *Leuctrabraueri* exhibited intraspecific haplotype variation. Key findings include uncovering cryptic diversity within the *Isoperlagrammatica* species complex and the discovery of a potentially new species of the genus *Leuctra*. Ultimately, these results provide a foundational basis for subsequent systematic analyses of population structuring and phylogenetic studies involving stoneflies in Europe.

## Supplementary Material

8FC226B5-0F04-5B49-8179-760CE4601D3F 10.3897/BDJ.12.e121398.suppl1 Supplementary material 1 Utilising public sequence databases to investigate genetic diversity of stoneflies in Medvednica Nature Park Data type Occurrences, phylogenetic File: oo_1013026.docx https://binary.pensoft.net/file/1013026 Dora Kermek, Nikola Pischiutta, Dora Hlebec, Ignac Sivec, Mladen Kučinić
