# Supplementary material for: Utilising public sequence databases to investigate genetic diversity of stoneflies in Medvednica Nature Park
Source: Biodivers Data J. 2024 Apr 18;12:e121398. doi: 10.3897/BDJ.12.e121398 (PMC11046089; doi:10.3897/BDJ.12.e121398)
Supplement: Supplementary material 1 — Utilising public sequence databases to investigate genetic diversity of stoneflies in Medvednica Nature Park [file bdj-12-e121398-s001.docx]

Supplementary Information for:

**Utilizing public sequence databases to investigate genetic diversity of stoneflies in Medvednica Nature Park**

Dora Kermek, Nikola Pischiutta, Dora Hlebec, Ignac Sivec, Mladen Kučinić

**Supplementary Table S1.** A list of sampling sites with their corresponding coordinates in the WGS coordinate system, along with a list of identified species.

**Supplementary Table S2.** *COI* sequences and haplotypes downloaded from BOLD database and used in phylogenetic analyses.

**Supplementary Table S3.** A summary of species delimitation obtained through the ASAP, ABGD, bPTP and BIN assignment for all analyzed species. For ASAP and ABGD methods, only the two partitions with the highest scores are shown.

**Supplementary Table S4.** Detailed results of the species delineation using ASAP (*p*-distance and Kimura 2-parameter [K80] distance ts/tv option) and ABGD (Kimura 2-parameter [K80] distance ts/tv option).

**Supplementary Table S5**. Ranges of intraspecific uncorrected pairwise distances (*p*-distances) within and between groups (G1–G3) of the species *Brachyptera seticornis*, as indicated on the phylogenetic tree and network (see Figures 2.A and 2.B). The maximum genetic distance within groups is highlighted in yellow, while the range of genetic distance between groups is shown in white.

**Supplementary Table S6**. Ranges of intraspecific uncorrected pairwise distances (*p*-distances) within and between groups (G1–G2) of the species *Leuctra braueri*, as indicated on the phylogenetic tree and network (see Figures 3.A and 3.B). The maximum genetic distance within groups is highlighted in yellow, while the range of genetic distance between groups is shown in white.

**Supplementary Table S7**. Ranges of intraspecific uncorrected pairwise distances (*p*-distances) within and between groups (G1–G4) of the species *Leuctra prima* and *Leuctra* sp. ZB, as indicated on the phylogenetic tree and network (see Figures 4.A and 4.B). The maximum genetic distance within groups is highlighted in yellow, while the range of genetic distance between groups is shown in white.

**Supplementary Table S8**. Ranges of intraspecific uncorrected pairwise distances (*p*-distances) within and between groups (G1–G6) of the species *Isoperla grammatica*, as indicated on the phylogenetic tree and network (see Figures 5.A and 5.B). The maximum genetic distance within groups is highlighted in yellow, while the range of genetic distance between groups is shown in white.

**Supplementary Table S1.** A list of sampling sites with their corresponding coordinates in the WGS coordinate system, along with a list of identified species. Morphological determinations include information on life stages, sex, and the number of individuals. Results of the DNA barcoding is accompanied by the BOLD IDs and BINs.

| **Sampling site** | | **Longitude (WGS)** | **Latitude (WGS)** | **Altitude** |
| --- | --- | --- | --- | --- |
| S1 | Medvednica, creek Kraljevec, lower | 15.947999 | 45.865729 | 378 |
| S2 | Medvednica, creek Kraljevec, middle | 15.942650 | 45.871876 | 496 |
| S3 | Medvednica, creek Kraljevec, Kraljičin Zdenac | 15.940083 | 45.876077 | 523 |
| S4 | Medvednica, creek Bliznec | 15.977430 | 45.878410 | 401 |
| S5 | Medvednica, creek Družinec | 15.895981 | 45.843275 | 294 |
| S6 | Medvednica, creek Jambrišakovo vrelo | 15.884428 | 45.858836 | 504 |
| S7 | Medvednica, creek Jelenja voda | 15.963550 | 45.917929 | 639 |
| S8 | Medvednica, creek Kraljevec, upper | 15.943486 | 45.883658 | 607 |

| **Family, species** | **Morphological determination**  Life stage, sex (Nr. Ind.) | **DNA barcoding**  BOLD ID, BIN | **Sampling sites** |
| --- | --- | --- | --- |
| Taeniopterygidae (Klapálek, 1905) | | | |
| *Brachyptera seticornis* (Klapálek, 1902) | Larva (6) | CROPL392-22, AAY5851 | S8 |
| Perlodidae (Klapálek, 1909) | | | |
| *Isoperla grammatica* (Poda, 1761) | Larva (5) | CROPL395-22, AER8749  CROPL396-22, AER8749  CROPL397-22, AER8749 | S5 |
| Leuctridae (Klapálek, 1905) | | | |
| *Leuctra braueri* (Kempny, 1898) | Adult, ♂ (30)  Adult, ♀ (33) | CROPL375-22, AES9453  CROPL376-22, AES9453  CROPL378-22, AES9453  CROPL379-22, AES9453  CROPL380-22, AES9453  CROPL385-22, AES9453  CROPL386-22, AES9453  CROPL387-22, AES9453  CROPL389-22, AES9453 | S1, S2, S3 |
| *Leuctra cingulata* (Kempny, 1899) | Adult, ♀ (4) | CROPL377-22, AAK9204 | S1 |
| *Leuctra nigra* (Olivier, 1811) | Adult, ♂ (1) | - | S4 |
| *Leuctra signifera* (Kempny, 1899) | Adult, ♂ (1) | - | S2 |
| *Leuctra prima* (Kempny, 1899) | Larva (2) | CROPL391-22, ACB1947 | S8 |
| *Leuctra* sp. ZB | Adult (1) | CROPL384-22, AES3404 | S1 |
| Perlidae (Latreille, 1802) | | | |
| *Perla pallida* (Guérin-Méneville, 1838.) | Larva (2) | CROPL390-22, AEB9929  CROPL383-22, AEB9929 | S4, S7 |
| Nemouridae (Newmann, 1853) | | | |
| *Protonemura auberti* (Illies, 1954) | Adult, ♂ (2) | CROPL381-22, AAH7653  CROPL382-22, AAH7653 | S7 |
| *Protonemura intricata* (Ris, 1902) | Adult, ♂ (2)  Adult, ♀ (1) | CROPL373-22, AAM9758  CROPL374-22, AAM9758 | S4 |
| *Protonemura nitida* (Pictet, 1836) | Adult, ♀ (1) | CROPL388-22, AED9051 | S1 |
| *Protonemura praecox* (Morton, 1894) | Larva (1) | CROPL393-22, AES9615 | S8 |
| *Nemoura sciurus* (Aubert, 1949) | Larva (9) | CROPL394-22, AED4647 | S6 |

**Supplementary Table S2.** *COI* sequences and haplotypes downloaded from BOLD database and used in phylogenetic analyses.

| **Species, haplotype** | **Number of sequences in a haplotype** | **BOLD-ID** | **Country** |
| --- | --- | --- | --- |
| ***Brachyptera seticornis*** | | | |
| BGMAY490-11 | 2 | BGMAY490-11 | Bulgaria |
|  |  | BGMAY502-11 | Serbia |
| BGMAY500-11 | 1 | BGMAY500-11 | Serbia |
| BGMAY501-11 | 1 | BGMAY501-11 | Serbia |
| CROPL014-21 | 1 | CROPL014-21 | Croatia, Medvednica |
| CROPL046-21 | 1 | CROPL046-21 | Croatia, Zrinska gora |
| CROPL049-21 | 1 | CROPL049-21 | Croatia, Zrinska gora |
| CROPL055-21 | 1 | CROPL055-21 | Croatia, Medvednica |
| CROPL139-21 | 2 | CROPL139-21 | Croatia, Papuk |
|  |  | CROPL149-21 | Croatia, Kaptol |
| GBMND82057-21 | 1 | GBMND82057-21 | Germany |
| GBMND82058-21 | 1 | GBMND82058-21 | Germany |
| GBMND82059-21 | 1 | GBMND82059-21 | Germany |
| GBMND82063-21 | 4 | GBMND82063-21 | Germany |
|  |  | GBMND82067-21 | Germany |
|  |  | GBMND82069-21 | Germany |
|  |  | GBMND82077-21 | Germany |
| GBMND82064-21 | 1 | GBMND82064-21 | Germany |
| GBMND82066-21 | 1 | GBMND82066-21 | Germany |
| GBMND82079-21 | 1 | GBMND82079-21 | Germany |
| GBMND82080-21 | 1 | GBMND82080-21 | Germany |
| GBMND82084-21 | 1 | GBMND82084-21 | Germany |
| GBMND82088-21 | 1 | GBMND82088-21 | Germany |
| GBMND82093-21 | 1 | GBMND82093-21 | Germany |
| FBAQU1304-12 | 33 | FBAQU1304-12 | Germany |
|  |  | GBEPT301-14 | Germany |
|  |  | GBMND82052-21 | Germany |
|  |  | GBMND82053-21 | Germany |
|  |  | GBMND82054-21 | Germany |
|  |  | GBMND82055-21 | Germany |
|  |  | GBMND82056-21 | Germany |
|  |  | CROPL392-22 | Croatia, Medvednica |
|  |  | GBMND82060-21 | Germany |
|  |  | GBMND82061-21 | Germany |
|  |  | GBMND82062-21 | Germany |
|  |  | GBMND82065-21 | Germany |
|  |  | GBMND82068-21 | Germany |
|  |  | GBMND82070-21 | Germany |
|  |  | GBMND82071-21 | Germany |
|  |  | GBMND82072-21 | Germany |
|  |  | GBMND82073-21 | Germany |
|  |  | GBMND82074-21 | Germany |
|  |  | GBMND82075-21 | Germany |
|  |  | GBMND82076-21 | Germany |
|  |  | GBMND82078-21 | Germany |
|  |  | GBMND82081-21 | Germany |
|  |  | GBMND82082-21 | Germany |
|  |  | GBMND82083-21 | Germany |
|  |  | GBMND82085-21 | Germany |
|  |  | GBMND82086-21 | Germany |
|  |  | GBMND82087-21 | Germany |
|  |  | GBMND82089-21 | Germany |
|  |  | GBMND82090-21 | Germany |
|  |  | GBMND82091-21 | Germany |
|  |  | GBMND82092-21 | Germany |
|  |  | GBMND82094-21 | Germany |
|  |  | PLEAA161-20 | Switzerland |
| ***Leuctra braueri*** | | | |
| FBAQU194-09 | 1 | FBAQU194-09 | Germany |
| FBAQU331-09 | 12 | FBAQU331-09 | Germany |
|  |  | GBEPT1008-14 | Germany |
|  |  | GBEPT2302-15 | Germany |
|  |  | GBEPT2359-15 | Germany |
|  |  | GBEPT2364-15 | Germany |
|  |  | GBEPT2371-15 | Germany |
|  |  | GBMIX1524-15 | Germany |
|  |  | GBMIX1528-15 | Germany |
|  |  | GBMIX1531-15 | Germany |
|  |  | GBMIX2475-15 | Germany |
|  |  | GBMIX2485-15 | Germany |
|  |  | GBMIX2465-15 | Germany |
| GBEPT2353-15 | 6 | GBEPT2353-15 | Germany |
|  |  | GBEPT2354-15 | Germany |
|  |  | GBEPT2355-15 | Germany |
|  |  | GBEPT2356-15 | Germany |
|  |  | GBEPT2357-15 | Germany |
|  |  | GBEPT2358-15 | Germany |
| GBMIX1667-15 | 5 | GBMIX1667-15 | Germany |
|  |  | INTAP035-17 | Austria |
|  |  | INTAP036-17 | Austria |
|  |  | INTAP037-17 | Austria |
|  |  | PLEAA096-20 | Switzerland |
| GBMIX2463-15 | 1 | GBMIX2463-15 | Germany |
| GBMNC47659-20 | 1 | GBMNC47659-20 | Italy |
| GBMNC47660-20 | 1 | GBMNC47660-20 | Italy |
| CROPL389-22 | 1 | CROPL389-22 | Croatia |
| CROPL379-22 | 1 | CROPL379-22 | Croatia |
| CROPL385-22 | 2 | CROPL385-22 | Croatia |
|  |  | CROPL375-22 | Croatia |
| CROPL387-22 | 1 | CROPL387-22 | Croatia |
| CROPL386-22 | 1 | CROPL386-22 | Croatia |
| CROPL376-22 | 1 | CROPL376-22 | Croatia |
| CROPL378-22 | 1 | CROPL378-22 | Croatia |
| GMGRI255-13 | 1 | GMGRI255-13 | Germany |
| GST167-15 | 1 | GST167-15 | Germany |
| INTAP038-17 | 1 | INTAP038-17 | Austria |
| PLEAA170-20 | 1 | PLEAA170-20 | Switzerland |
| PLEAA413-20 | 1 | PLEAA413-20 | Switzerland |
| ***Leuctra prima* / *Leuctra* sp. ZB** | | | |
| CROPL248-21 | 1 | CROPL248-21 | Croatia, Žumberak |
| CROPL391-22 | 2 | CROPL391-22 | Croatia, Medvednica |
|  |  | CROPL016-21 | Croatia, Medvednica |
| CROPL384-22 | 1 | CROPL384-22 | Croatia, Medvednica |
| CROPL003-21 | 1 | CROPL003-21 | Croatia, Gorski kotar |
| CROPL023-21 | 1 | CROPL023-21 | Croatia, Medvednica |
| FBAQU1315-12 | 1 | FBAQU1315-12 | Germany |
| GST207-15 | 6 | GST207-15 | Germany |
|  |  | GST209-15 | Germany |
|  |  | GST212-15 | Germany |
|  |  | GST214-15 | Germany |
|  |  | GST218-15 | Germany |
|  |  | GST220-15 | Germany |
| GST208-15 | 5 | GST208-15 | Germany |
|  |  | GST210-15 | Germany |
|  |  | GST215-15 | Germany |
|  |  | GST217-15 | Germany |
|  |  | GST219-15 | Germany |
| PLEAA205-20 | 1 | PLEAA205-20 | Switzerland |
| PLEAA382-20 | 1 | PLEAA382-20 | Switzerland |
| ***Isoperla grammatica*** | | | |
| CROPL154-21 | 1 | CROPL154-21 | Croatia, Slavonija |
| CROPL205-21 | 1 | CROPL205-21 | Croatia, Međimurje |
| CROPL265-21 | 1 | CROPL265-21 | Croatia, Slavonija |
| CROPL276-21 | 2 | CROPL276-21 | Croatia, Slavonija |
|  |  | CROPL342-21 | Croatia, Slavonija |
| CROPL318-21 | 1 | CROPL318-21 | Slovenia, Ljubljana |
| CROPL338-21 | 1 | CROPL338-21 | Croatia, Slavonija |
| CROPL292-21 | 1 | CROPL292-21 | Croatia, Slavonija |
| CROPL395-22 | 2 | CROPL395-22 | Croatia, Medvednica |
|  |  | CROPL396-22 | Croatia, Medvednica |
| CROPL397-22 | 1 | CROPL397-22 | Croatia, Medvednica |
| FBAQU007-09 | 1 | FBAQU007-09 | Germany |
| FIEPT075-11 | 1 | FIEPT075-11 | Finland |
| FIEPT076-11 | 1 | FIEPT076-11 | Finland |
| FIEPT078-11 | 1 | FIEPT078-11 | Finland |
| FIEPT079-11 | 1 | FIEPT079-11 | Finland |
| GBMNC47877-20 | 1 | GBMNC47877-20 | Italy |
| GBMNC47878-20 | 1 | GBMNC47878-20 | Italy |
| GBMNC47879-20 | 3 | GBMNC47879-20 | Italy |
|  |  | GBMNC47880-20 | Italy |
|  |  | GBMNC47881-20 | Italy |
| GBMNC47882-20 | 1 | GBMNC47882-20 | Italy |
| GBMNC47883-20 | 1 | GBMNC47883-20 | Italy |
| GBMNC47884-20 | 1 | GBMNC47884-20 | Italy |
| GBMNC47885-20 | 1 | GBMNC47885-20 | Italy |
| GBMNC47886-20 | 2 | GBMNC47886-20 | Italy |
|  |  | GBMNC47887-20 | Italy |
| GBMNC47888-20 | 2 | GBMNC47888-20 | Italy |
|  |  | GBMNC47889-20 | Italy |
| GBMNC47890-20 | 1 | GBMNC47890-20 | Italy |
| GBMNC47891-20 | 2 | GBMNC47891-20 | Italy |
|  |  | GBMNC47892-20 | Italy |
| IBIPP003-19 | 6 | IBIPP003-19 | Portugal |
|  |  | IBIPP035-20 | Portugal |
|  |  | IBIPP036-20 | Portugal |
|  |  | IBIPP053-20 | Portugal |
|  |  | IBIPP058-20 | Portugal |
|  |  | IBIPP085-20 | Portugal |
| IBIPP059-20 | 1 | IBIPP059-20 | Portugal |
| IBIPP084-20 | 1 | IBIPP084-20 | Portugal |
| IBIPP034-20 | 1 | IBIPP034-20 | Portugal |
| IBIPP104-20 | 1 | IBIPP104-20 | Portugal |
| INTAP172-17 | 1 | INTAP172-17 | Austria |
| PLEAA243-20 | 1 | PLEAA243-20 | Switzerland |
| PLEAA324-20 | 1 | PLEAA324-20 | Switzerland |

**Supplementary Table S3.** A summary of species delimitation obtained through the ASAP, ABGD, bPTP and BIN assignment for all analyzed species. For ASAP and ABGD methods, only the two partitions with the highest scores are shown.

| **Species** | **Species delimitation method** | **Score** | **Number of putative species groups** |
| --- | --- | --- | --- |
| *Brachyptera seticornis* | ASAP *p*-distance | asap-score = 1.50 | 3 |
|  |  | asap-score = 1.50 | 4 |
|  | ASAP Kimura (K80) | asap-score = 1.50 | 3 |
|  |  | asap-score = 1.50 | 4 |
|  | ABDG Kimura (K80) | P = 4.64^-3^ | 1 |
|  |  | P = 2.78^-3^ | 2 |
|  | bPTP | / | 1 |
|  | BIN | / | 1 (AAY5851) |
| *Leuctra braueri* | ASAP *p*-distance | asap-score = 2.50 | 4 |
|  |  | asap-score = 4.00 | 5 |
|  | ASAP Kimura (K80) | asap-score = 3.00 | 3 |
|  |  | asap-score = 3.50 | 4 |
|  | ABDG Kimura (K80) | P = 1.67^-3^ to  P = 1.29^-2^ | 2 |
|  | bPTP | / | 1 |
|  | BIN | / | 2 (AAJ2415, AES9453) |
| *Leuctra prima /*  *Leuctra sp. ZB* | ASAP *p*-distance | asap-score = 1.50 | 2 |
|  |  | asap-score = 2.50 | 4 |
|  | ASAP Kimura (K80) | asap-score = 1.50 | 2 |
|  |  | asap-score = 2.50 | 4 |
|  | ABDG Kimura (K80) | P = 2.15^-2^ to  P = 7.74^-3^ | 2 |
|  |  | P = 4.64^-3^ | 4 |
|  | bPTP | / | 3 |
|  | BIN | / | 2 (AEE0093, ACB1947) |
| *Isoperla grammatica* | ASAP *p*-distance | asap-score = 2.00 | 6 |
|  |  | asap-score = 3.50 | 3 |
|  | ASAP Kimura (K80) | asap-score = 2.50 | 6 |
|  |  | asap-score = 3.00 | 3 |
|  | ABDG Kimura (K80) | P = 2.78^-3^ | 6 |
|  |  | P = 7.74^-3^,  P = 4.64^-3^ | 5 |
|  | bPTP | / | 3 |
|  | BIN | / | 7 (AAY9655, AEH6396, AEG4373, AEC9627, ACJ0709, AAK4351, AER8749) |

**Supplementary Table S4.** Detailed results of the species delineation using ASAP (*p*-distance and Kimura 2-parameter [K80] distance ts/tv option) and ABGD (Kimura 2-parameter [K80] distance ts/tv option).

| **Method, species, partitions, and groups** |
| --- |
| **ABGD** |
| ***Brachyptera seticornis*** |
| Initial Partition with prior maximal distance P=1.00e-03 ; Barcode gap distance = 0.001 Distance K80 Kimura MinSlope=1.500000  **Group[ 1 ] n: 2 ;**id: BGMAY490-11 BGMAY502-11  **Group[ 2 ] n: 1 ;**id: BGMAY500-11  **Group[ 3 ] n: 1 ;**id: BGMAY501-11  **Group[ 4 ] n: 1 ;**id: CROPL014-21  **Group[ 5 ] n: 1 ;**id: CROPL046-21  **Group[ 6 ] n: 1 ;**id: CROPL049-21  **Group[ 7 ] n: 1 ;**id: CROPL055-21  **Group[ 8 ] n: 2 ;**id: CROPL139-21 CROPL149-21  **Group[ 9 ] n: 33 ;**id: FBAQU1304-12 GBEPT301-14 GBMND82052-21 GBMND82053-21 GBMND82054-21 GBMND82055-21 GBMND82056-21 CROPL392-22 GBMND82060-21 GBMND82061-21 GBMND82062-21 GBMND82065-21 GBMND82068-21 GBMND82070-21 GBMND82071-21 GBMND82072-21 GBMND82073-21 GBMND82074-21 GBMND82075-21 GBMND82076-21 GBMND82078-21 GBMND82081-21 GBMND82082-21 GBMND82083-21 GBMND82085-21 GBMND82086-21 GBMND82087-21 GBMND82089-21 GBMND82090-21 GBMND82091-21 GBMND82092-21 GBMND82094-21 PLEAA161-20  **Group[ 10 ] n: 1 ;**id: GBMND82057-21  **Group[ 11 ] n: 1 ;**id: GBMND82058-21  **Group[ 12 ] n: 1 ;**id: GBMND82059-21 **Group[ 13 ] n: 4 ;**id: GBMND82063-21 GBMND82067-21 GBMND82069-21 GBMND82077-21 **Group[ 14 ] n: 1 ;**id: GBMND82064-21  **Group[ 15 ] n: 1 ;**id: GBMND82066-21  **Group[ 16 ] n: 1 ;**id: GBMND82079-21  **Group[ 17 ] n: 1 ;**id: GBMND82080-21  **Group[ 18 ] n: 1 ;**id: GBMND82084-21  **Group[ 19 ] n: 1 ;**id: GBMND82088-21  **Group[ 20 ] n: 1 ;**id: GBMND82093-21  Partitions with prior maximal distances P=1.67e-03, P=2.78e-03 Distance K80 Kimura MinSlope=1.500000  **Group[ 1 ] n: 5 ;**id: BGMAY490-11 BGMAY500-11 BGMAY501-11 BGMAY502-11 CROPL049-21 **Group[ 2 ] n: 3 ;**id: CROPL014-21 CROPL139-21 CROPL149-21  **Group[ 3 ] n: 1 ;**id: CROPL046-21  **Group[ 4 ] n: 47 ;**id: CROPL055-21 FBAQU1304-12 GBEPT301-14 GBMND82052-21 GBMND82053-21 GBMND82054-21 GBMND82055-21 GBMND82056-21 GBMND82057-21 CROPL392-22 GBMND82058-21 GBMND82059-21 GBMND82060-21 GBMND82061-21 GBMND82062-21 GBMND82063-21 GBMND82064-21 GBMND82065-21 GBMND82066-21 GBMND82067-21 GBMND82068-21 GBMND82069-21 GBMND82070-21 GBMND82071-21 GBMND82072-21 GBMND82073-21 GBMND82074-21 GBMND82075-21 GBMND82076-21 GBMND82077-21 GBMND82078-21 GBMND82080-21 GBMND82081-21 GBMND82082-21 GBMND82083-21 GBMND82084-21 GBMND82085-21 GBMND82086-21 GBMND82087-21 GBMND82088-21 GBMND82089-21 GBMND82090-21 GBMND82091-21 GBMND82092-21 GBMND82093-21 GBMND82094-21 PLEAA161-20  **Group[ 5 ] n: 1 ;**id: GBMND82079-21  Initial Partitions with prior maximal distances P=4.64e-03, P=7.74e-03, P=1.29e-02 ; Barcode gap distance = 0.013 Distance K80 Kimura MinSlope=1.500000  **Group[ 1 ] n: 54 ;**id: BGMAY490-11 BGMAY500-11 BGMAY501-11 BGMAY502-11 CROPL046-21 CROPL049-21 CROPL055-21 FBAQU1304-12 GBEPT301-14 GBMND82052-21 GBMND82053-21 GBMND82054-21 GBMND82055-21 GBMND82056-21 GBMND82057-21 CROPL392-22 GBMND82058-21 GBMND82059-21 GBMND82060-21 GBMND82061-21 GBMND82062-21 GBMND82063-21 GBMND82064-21 GBMND82065-21 GBMND82066-21 GBMND82067-21 GBMND82068-21 GBMND82069-21 GBMND82070-21 GBMND82071-21 GBMND82072-21 GBMND82073-21 GBMND82074-21 GBMND82075-21 GBMND82076-21 GBMND82077-21 GBMND82078-21 GBMND82079-21 GBMND82080-21 GBMND82081-21 GBMND82082-21 GBMND82083-21 GBMND82084-21 GBMND82085-21 GBMND82086-21 GBMND82087-21 GBMND82088-21 GBMND82089-21 GBMND82090-21 GBMND82091-21 GBMND82092-21 GBMND82093-21 GBMND82094-21 PLEAA161-20  **Group[ 2 ] n: 3 ;**id: CROPL014-21 CROPL139-21 CROPL149-21 |
| ***Leuctra braueri*** |
| Initial Partition with prior maximal distance P=1.00e-03 ; Barcode gap distance = 0.001 Distance K80 Kimura MinSlope=1.500000  **Group[ 1 ] n: 1 ;**id: FBAQU194-09  **Group[ 2 ] n: 13 ;**id: FBAQU331-09 GBEPT1008-14 GBEPT2302-15 GBEPT2359-15 GBEPT2364-15 GBEPT2371-15 GBMIX1524-15 GBMIX1528-15 GBMIX1531-15 GBMIX2465-15 GBMIX2475-15 GBMIX2485-15 GBMNC47659-15  **Group[ 3 ] n: 6 ;**id: GBEPT2353-15 GBEPT2354-15 GBEPT2355-15 GBEPT2356-15 GBEPT2357-15 GBEPT2358-15  **Group[ 4 ] n: 7 ;**id: GBMIX1667-15 INTAP035-17 INTAP036-17 INTAP037-17 PLEAA096-20 PLEAA170-20 PLEAA413-20  **Group[ 5 ] n: 1 ;**id: GBMIX2463-15  **Group[ 6 ] n: 1 ;**id: GBMNC47660-20  **Group[ 7 ] n: 1 ;**id: CROPL389-22  **Group[ 8 ] n: 1 ;**id: CROPL379-22  **Group[ 9 ] n: 3 ;**id: CROPL385-22 CROPL375-22 CROPL380-22  **Group[ 10 ] n: 1 ;**id: CROPL387-22  **Group[ 11 ] n: 1 ;**id: CROPL386-22  **Group[ 12 ] n: 1 ;**id: CROPL376-22  **Group[ 13 ] n: 1 ;**id: CROPL378-22  **Group[ 14 ] n: 1 ;**id: GMGRI255-13  **Group[ 15 ] n: 1 ;**id: GST167-15  **Group[ 16 ] n: 1 ;**id: INTAP038-17  Initial Partitions with prior maximal distances P=1.67e-03, P=2.78e-03, P=4.64e-03, P=7.74e-03, P=1.29e-02; Barcode gap distance = 0.013 Distance K80 Kimura MinSlope=1.500000  **Group[ 1 ] n: 32 ;**id: FBAQU194-09 FBAQU331-09 GBEPT1008-14 GBEPT2302-15 GBEPT2359-15 GBEPT2364-15 GBEPT2371-15 GBMIX1524-15 GBMIX1528-15 GBMIX1531-15 GBMIX2465-15 GBMIX2475-15 GBMIX2485-15 GBMNC47659-15 GBEPT2353-15 GBEPT2354-15 GBEPT2355-15 GBEPT2356-15 GBEPT2357-15 GBEPT2358-15 GBMIX1667-15 INTAP035-17 INTAP036-17 INTAP037-17 PLEAA096-20 PLEAA170-20 PLEAA413-20 GBMIX2463-15 GMGRI255-13 GBMNC47660-20 INTAP038-17 GST167-15  **Group[ 2 ] n: 9 ;**id: CROPL389-22 CROPL379-22 CROPL385-22 CROPL387-22 CROPL386-22 CROPL375-22 CROPL376-22 CROPL378-22 CROPL380-22 |
| ***Isoperla grammatica*** |
| Partition with prior maximal distance P=1.00e-03 Distance K80 Kimura MinSlope=1.500000  **Group[ 1 ] n: 7 ;**id: CROPL154-21 CROPL205-21 CROPL265-21 CROPL276-21 CROPL292-21 CROPL338-21 CROPL342-21  **Group[ 2 ] n: 4 ;**id: CROPL318-21 FBAQU007-09 GBMNC47877-20 INTAP172-17  **Group[ 3 ] n: 3 ;**id: CROPL395-22 CROPL396-22 CROPL397-22  **Group[ 4 ] n: 1 ;**id: FIEPT075-11  **Group[ 5 ] n: 15 ;**id: GBMNC47878-20 GBMNC47879-20 GBMNC47880-20 GBMNC47881-20 GBMNC47882-20 GBMNC47883-20 GBMNC47884-20 GBMNC47885-20 GBMNC47886-20 GBMNC47887-20 GBMNC47888-20 GBMNC47889-20 GBMNC47890-20 GBMNC47891-20 GBMNC47892-20  **Group[ 6 ] n: 1 ;**id: FIEPT076-11  **Group[ 7 ] n: 1 ;**id: FIEPT078-11  **Group[ 8 ] n: 1 ;**id: FIEPT079-11  **Group[ 9 ] n: 6 ;**id: IBIPP003-19 IBIPP035-20 IBIPP036-20 IBIPP053-20 IBIPP058-20 IBIPP085-20  **Group[ 10 ] n: 1 ;**id: IBIPP034-20  **Group[ 11 ] n: 1 ;**id: IBIPP059-20  **Group[ 12 ] n: 1 ;**id: IBIPP084-20  **Group[ 13 ] n: 1 ;**id: IBIPP104-20  **Group[ 14 ] n: 1 ;**id: PLEAA243-20  **Group[ 15 ] n: 1 ;**id: PLEAA324-20  Partitions with prior maximal distances P=1.67e-03, P=2.78e-03 Distance K80 Kimura MinSlope=1.500000  **Group[ 1 ] n: 7 ;**id: CROPL154-21 CROPL205-21 CROPL265-21 CROPL276-21 CROPL292-21 CROPL338-21 CROPL342-21  **Group[ 2 ] n: 4 ;**id: CROPL318-21 FBAQU007-09 GBMNC47877-20 INTAP172-17  **Group[ 3 ] n: 3 ;**id: CROPL395-22 CROPL396-22 CROPL397-22  **Group[ 4 ] n: 6 ;**id: FIEPT075-11 FIEPT076-11 FIEPT078-11 FIEPT079-11 PLEAA243-20 PLEAA324-20  **Group[ 5 ] n: 15 ;**id: GBMNC47878-20 GBMNC47879-20 GBMNC47880-20 GBMNC47881-20 GBMNC47882-20 GBMNC47883-20 GBMNC47884-20 GBMNC47885-20 GBMNC47886-20 GBMNC47887-20 GBMNC47888-20 GBMNC47889-20 GBMNC47890-20 GBMNC47891-20 GBMNC47892-20  **Group[ 6 ] n: 10 ;**id: IBIPP003-19 IBIPP034-20 IBIPP035-20 IBIPP036-20 IBIPP053-20 IBIPP058-20 IBIPP059-20 IBIPP084-20 IBIPP085-20 IBIPP104-20  Partitions with prior maximal distances P=4.64e-03, P=7.74e-03 Distance K80 Kimura MinSlope=1.500000  **Group[ 1 ] n: 11 ;**id: CROPL154-21 CROPL205-21 CROPL265-21 CROPL276-21 CROPL292-21 CROPL338-21 CROPL342-21 CROPL318-21 FBAQU007-09 GBMNC47877-20 INTAP172-17 **Group[ 2 ] n: 3 ;**id: CROPL395-22 CROPL396-22 CROPL397-22  **Group[ 3 ] n: 6 ;**id: FIEPT075-11 FIEPT076-11 FIEPT078-11 FIEPT079-11 PLEAA243-20 PLEAA324-20  **Group[ 4 ] n: 15 ;**id: GBMNC47878-20 GBMNC47879-20 GBMNC47880-20 GBMNC47881-20 GBMNC47882-20 GBMNC47883-20 GBMNC47884-20 GBMNC47885-20 GBMNC47886-20 GBMNC47887-20 GBMNC47888-20 GBMNC47889-20 GBMNC47890-20 GBMNC47891-20 GBMNC47892-20  **Group[ 5 ] n: 10 ;**id: IBIPP003-19 IBIPP034-20 IBIPP035-20 IBIPP036-20 IBIPP053-20 IBIPP058-20 IBIPP059-20 IBIPP084-20 IBIPP085-20 IBIPP104-20 |
| ***Leuctra prima* / *Leuctra* sp. ZB** |
| Initial Partition with prior maximal distance P=1.00e-03 ; Barcode gap distance = 0.001 Distance K80 Kimura MinSlope=1.500000  **Group[ 1 ] n: 1 ;**id: CROPL248-21  **Group[ 2 ] n: 2 ;**id: CROPL391-22 CROPL016-21  **Group[ 3 ] n: 1 ;**id: CROPL384-22  **Group[ 4 ] n: 1 ;**id: CROPL003-21  **Group[ 5 ] n: 1 ;**id: CROPL023-21  **Group[ 6 ] n: 1 ;**id: FBAQU1315-12  **Group[ 7 ] n: 6 ;**id: GST207-15 GST209-15 GST212-15 GST214-15 GST218-15 GST220-15  **Group[ 8 ] n: 5 ;**id: GST208-15 GST210-15 GST215-15 GST217-15 GST219-15  **Group[ 9 ] n: 1 ;**id: PLEAA205-20  **Group[ 10 ] n: 1 ;**id: PLEAA382-20  Initial Partition with prior maximal distance P=1.67e-03 ; Barcode gap distance = 0.005 Distance K80 Kimura MinSlope=1.500000  **Group[ 1 ] n: 1 ;**id: CROPL248-21  **Group[ 2 ] n: 13 ;**id: CROPL391-22 CROPL016-21 GST207-15 GST208-15 GST209-15 GST210-15 GST212-15 GST214-15 GST215-15 GST217-15 GST218-15 GST219-15 GST220-15  **Group[ 3 ] n: 1 ;**id: CROPL384-22  **Group[ 4 ] n: 1 ;**id: CROPL003-21  **Group[ 5 ] n: 1 ;**id: CROPL023-21  **Group[ 6 ] n: 1 ;**id: FBAQU1315-12  **Group[ 7 ] n: 2 ;**id: PLEAA205-20 PLEAA382-20  Initial Partition with prior maximal distance P=2.78e-03 ; Barcode gap distance = 0.005 Distance K80 Kimura MinSlope=1.500000  **Group[ 1 ] n: 1 ;**id: CROPL248-21  **Group[ 2 ] n: 13 ;**id: CROPL391-22 CROPL016-21 GST207-15 GST208-15 GST209-15 GST210-15 GST212-15 GST214-15 GST215-15 GST217-15 GST218-15 GST219-15 GST220-15  **Group[ 3 ] n: 1 ;**id: CROPL384-22  **Group[ 4 ] n: 1 ;**id: CROPL003-21  **Group[ 5 ] n: 1 ;**id: CROPL023-21  **Group[ 6 ] n: 1 ;**id: FBAQU1315-12  **Group[ 7 ] n: 2 ;**id: PLEAA205-20 PLEAA382-20  Initial Partition with prior maximal distance P=4.64e-03 ; Barcode gap distance = 0.023 Distance K80 Kimura MinSlope=1.500000  **Group[ 1 ] n: 1 ;**id: CROPL248-21  **Group[ 2 ] n: 16 ;**id: CROPL391-22 CROPL003-21 CROPL016-21 CROPL023-21 FBAQU1315-12 GST207-15 GST208-15 GST209-15 GST210-15 GST212-15 GST214-15 GST215-15 GST217-15 GST218-15 GST219-15 GST220-15  **Group[ 3 ] n: 1 ;**id: CROPL384-22  **Group[ 4 ] n: 2 ;**id: PLEAA205-20 PLEAA382-20  Initial Partitions with prior maximal distances P=1.29e-02, P=2.15e-02, P=3.59e-02, P=5.99e-02, P=1.00e-01; Barcode gap distance = 0.085 Distance K80 Kimura MinSlope=1.500000  **Group[ 1 ] n: 2 ;**id: CROPL248-21 CROPL384-22 **Group[ 2 ] n: 18 ;**id: CROPL391-22 CROPL003-21 CROPL016-21 CROPL023-21 FBAQU1315-12 GST207-15 GST208-15 GST209-15 GST210-15 GST212-15 GST214-15 GST215-15 GST217-15 GST218-15 GST219-15 GST220-15 PLEAA205-20 PLEAA382-20 |
| **ASAP Kimura (K80)** |
| ***Brachyptera seticornis*** |
| Partition 1  Score: 1  Proba: 3.357472e-02  nb groups:3 (2)  Group[ 1 ] n: 6 ;id: BGMAY490-11 BGMAY502-11 CROPL049-21 BGMAY500-11 BGMAY501-11 CROPL046-21  Group[ 2 ] n: 48 ;id: CROPL055-21 FBAQU1304-12 GBEPT301-14 GBMND82052-21 GBMND82053-21 GBMND82054-21 GBMND82055-21 GBMND82056-21 CROPL392-22 GBMND82060-21 GBMND82061-21 GBMND82062-21 GBMND82065-21 GBMND82068-21 GBMND82070-21 GBMND82071-21 GBMND82072-21 GBMND82073-21 GBMND82074-21 GBMND82075-21 GBMND82076-21 GBMND82078-21 GBMND82081-21 GBMND82082-21 GBMND82083-21 GBMND82085-21 GBMND82086-21 GBMND82087-21 GBMND82089-21 GBMND82090-21 GBMND82091-21 GBMND82092-21 GBMND82094-21 PLEAA161-20 GBMND82057-21 GBMND82058-21 GBMND82063-21 GBMND82067-21 GBMND82069-21 GBMND82077-21 GBMND82064-21 GBMND82080-21 GBMND82084-21 GBMND82088-21 GBMND82093-21 GBMND82059-21 GBMND82066-21 GBMND82079-21  Group[ 3 ] n: 3 ;id: CROPL014-21 CROPL139-21 CROPL149-21  Partition 2  Score: 2  Proba: 2.874251e-01  nb groups:5 (4)  Group[ 1 ] n: 5 ;id: BGMAY490-11 BGMAY502-11 CROPL049-21 BGMAY500-11 BGMAY501-11  Group[ 2 ] n: 3 ;id: CROPL014-21 CROPL139-21 CROPL149-21  Group[ 3 ] n: 1 ;id: CROPL046-21  Group[ 4 ] n: 47 ;id: CROPL055-21 FBAQU1304-12 GBEPT301-14 GBMND82052-21 GBMND82053-21 GBMND82054-21 GBMND82055-21 GBMND82056-21 CROPL392-22 GBMND82060-21 GBMND82061-21 GBMND82062-21 GBMND82065-21 GBMND82068-21 GBMND82070-21 GBMND82071-21 GBMND82072-21 GBMND82073-21 GBMND82074-21 GBMND82075-21 GBMND82076-21 GBMND82078-21 GBMND82081-21 GBMND82082-21 GBMND82083-21 GBMND82085-21 GBMND82086-21 GBMND82087-21 GBMND82089-21 GBMND82090-21 GBMND82091-21 GBMND82092-21 GBMND82094-21 PLEAA161-20 GBMND82057-21 GBMND82058-21 GBMND82063-21 GBMND82067-21 GBMND82069-21 GBMND82077-21 GBMND82064-21 GBMND82080-21 GBMND82084-21 GBMND82088-21 GBMND82093-21 GBMND82059-21 GBMND82066-21  Group[ 5 ] n: 1 ;id: GBMND82079-21  Partition 3  Score: 3  Proba: 5.648703e-01  nb groups:4 (3)  Group[ 1 ] n: 5 ;id: BGMAY490-11 BGMAY502-11 CROPL049-21 BGMAY500-11 BGMAY501-11  Group[ 2 ] n: 1 ;id: CROPL046-21  Group[ 3 ] n: 3 ;id: CROPL014-21 CROPL139-21 CROPL149-21  Group[ 4 ] n: 48 ;id: CROPL055-21 FBAQU1304-12 GBEPT301-14 GBMND82052-21 GBMND82053-21 GBMND82054-21 GBMND82055-21 GBMND82056-21 CROPL392-22 GBMND82060-21 GBMND82061-21 GBMND82062-21 GBMND82065-21 GBMND82068-21 GBMND82070-21 GBMND82071-21 GBMND82072-21 GBMND82073-21 GBMND82074-21 GBMND82075-21 GBMND82076-21 GBMND82078-21 GBMND82081-21 GBMND82082-21 GBMND82083-21 GBMND82085-21 GBMND82086-21 GBMND82087-21 GBMND82089-21 GBMND82090-21 GBMND82091-21 GBMND82092-21 GBMND82094-21 PLEAA161-20 GBMND82057-21 GBMND82058-21 GBMND82063-21 GBMND82067-21 GBMND82069-21 GBMND82077-21 GBMND82064-21 GBMND82080-21 GBMND82084-21 GBMND82088-21 GBMND82093-21 GBMND82059-21 GBMND82066-21 GBMND82079-21 |
| ***Leuctra braueri*** |
| Partition 1  Score: 1  Proba: 3.849057e-02  nb groups:2 (1)  Group[ 1 ] n: 32 ;id: FBAQU194-09 FBAQU331-09 GBEPT1008-14 GBEPT2302-15 GBEPT2359-15 GBEPT2364-15 GBEPT2371-15 GBMIX1524-15 GBMIX1528-15 GBMIX1531-15 GBMIX2465-15 GBMIX2475-15 GBMIX2485-15 GBMNC47659-15 GBEPT2353-15 GBEPT2354-15 GBEPT2355-15 GBEPT2356-15 GBEPT2357-15 GBEPT2358-15 GBMIX1667-15 INTAP035-17 INTAP036-17 INTAP037-17 PLEAA096-20 PLEAA170-20 PLEAA413-20 GBMIX2463-15 GMGRI255-13 GBMNC47660-20 INTAP038-17 GST167-15  Group[ 2 ] n: 9 ;id: CROPL389-22 CROPL379-22 CROPL385-22 CROPL375-22 CROPL380-22 CROPL378-22 CROPL386-22 CROPL376-22 CROPL387-22    Partition 2  Score: 2  Proba: 6.566866e-01  nb groups:3 (2)  Group[ 1 ] n: 31 ;id: FBAQU194-09 FBAQU331-09 GBEPT1008-14 GBEPT2302-15 GBEPT2359-15 GBEPT2364-15 GBEPT2371-15 GBMIX1524-15 GBMIX1528-15 GBMIX1531-15 GBMIX2465-15 GBMIX2475-15 GBMIX2485-15 GBMNC47659-15 GBEPT2353-15 GBEPT2354-15 GBEPT2355-15 GBEPT2356-15 GBEPT2357-15 GBEPT2358-15 GBMIX1667-15 INTAP035-17 INTAP036-17 INTAP037-17 PLEAA096-20 PLEAA170-20 PLEAA413-20 GBMIX2463-15 GMGRI255-13 GBMNC47660-20 INTAP038-17  Group[ 2 ] n: 1 ;id: GST167-15  Group[ 3 ] n: 9 ;id: CROPL389-22 CROPL379-22 CROPL385-22 CROPL375-22 CROPL380-22 CROPL378-22 CROPL386-22 CROPL376-22 CROPL387-22  Partition 3  Score: 3  Proba: 7.265469e-01  nb groups:4 (3)  Group[ 1 ] n: 31 ;id: FBAQU194-09 FBAQU331-09 GBEPT1008-14 GBEPT2302-15 GBEPT2359-15 GBEPT2364-15 GBEPT2371-15 GBMIX1524-15 GBMIX1528-15 GBMIX1531-15 GBMIX2465-15 GBMIX2475-15 GBMIX2485-15 GBMNC47659-15 GBEPT2353-15 GBEPT2354-15 GBEPT2355-15 GBEPT2356-15 GBEPT2357-15 GBEPT2358-15 GBMIX1667-15 INTAP035-17 INTAP036-17 INTAP037-17 PLEAA096-20 PLEAA170-20 PLEAA413-20 GBMIX2463-15 GMGRI255-13 GBMNC47660-20 INTAP038-17  Group[ 2 ] n: 1 ;id: CROPL389-22  Group[ 3 ] n: 8 ;id: CROPL379-22 CROPL385-22 CROPL375-22 CROPL380-22 CROPL378-22 CROPL386-22 CROPL376-22 CROPL387-22  Group[ 4 ] n: 1 ;id: GST167-15 |
| ***Isoperla grammatica*** |
| Partition 1  Score: 1  Proba: 2.495010e-01  nb groups:6 (5)  Group[ 1 ] n: 7 ;id: CROPL154-21 CROPL205-21 CROPL265-21 CROPL338-21 CROPL292-21 CROPL276-21 CROPL342-21  Group[ 2 ] n: 4 ;id: CROPL318-21 GBMNC47877-20 FBAQU007-09 INTAP172-17  Group[ 3 ] n: 3 ;id: CROPL395-22 CROPL396-22 CROPL397-22  Group[ 4 ] n: 6 ;id: FIEPT075-11 PLEAA243-20 FIEPT076-11 FIEPT078-11 FIEPT079-11 PLEAA324-20  Group[ 5 ] n: 10 ;id: IBIPP003-19 IBIPP035-20 IBIPP036-20 IBIPP053-20 IBIPP058-20 IBIPP085-20 IBIPP059-20 IBIPP104-20 IBIPP034-20 IBIPP084-20  Group[ 6 ] n: 15 ;id: GBMNC47878-20 GBMNC47879-20 GBMNC47880-20 GBMNC47881-20 GBMNC47886-20 GBMNC47887-20 GBMNC47888-20 GBMNC47889-20 GBMNC47882-20 GBMNC47890-20 GBMNC47891-20 GBMNC47892-20 GBMNC47883-20 GBMNC47884-20 GBMNC47885-20  Partition 2  Score: 2  Proba: 6.367265e-01  nb groups:3 (2)  Group[ 1 ] n: 27 ;id: CROPL154-21 CROPL205-21 CROPL265-21 CROPL338-21 CROPL292-21 CROPL276-21 CROPL342-21 CROPL318-21 GBMNC47877-20 FBAQU007-09 INTAP172-17 FIEPT075-11 PLEAA243-20 FIEPT076-11 FIEPT078-11 FIEPT079-11 PLEAA324-20 IBIPP003-19 IBIPP035-20 IBIPP036-20 IBIPP053-20 IBIPP058-20 IBIPP085-20 IBIPP059-20 IBIPP104-20 IBIPP034-20 IBIPP084-20  Group[ 2 ] n: 3 ;id: CROPL395-22 CROPL396-22 CROPL397-22  Group[ 3 ] n: 15 ;id: GBMNC47878-20 GBMNC47879-20 GBMNC47880-20 GBMNC47881-20 GBMNC47886-20 GBMNC47887-20 GBMNC47888-20 GBMNC47889-20 GBMNC47882-20 GBMNC47890-20 GBMNC47891-20 GBMNC47892-20 GBMNC47883-20 GBMNC47884-20 GBMNC47885-20  Partition 3  Score: 3  Proba: 7.105788e-01  nb groups:7 (6)  Group[ 1 ] n: 7 ;id: CROPL154-21 CROPL205-21 CROPL265-21 CROPL338-21 CROPL292-21 CROPL276-21 CROPL342-21  Group[ 2 ] n: 2 ;id: CROPL318-21 GBMNC47877-20  Group[ 3 ] n: 2 ;id: FBAQU007-09 INTAP172-17  Group[ 4 ] n: 3 ;id: CROPL395-22 CROPL396-22 CROPL397-22  Group[ 5 ] n: 6 ;id: FIEPT075-11 PLEAA243-20 FIEPT076-11 FIEPT078-11 FIEPT079-11 PLEAA324-20  Group[ 6 ] n: 15 ;id: GBMNC47878-20 GBMNC47879-20 GBMNC47880-20 GBMNC47881-20 GBMNC47886-20 GBMNC47887-20 GBMNC47888-20 GBMNC47889-20 GBMNC47882-20 GBMNC47890-20 GBMNC47891-20 GBMNC47892-20 GBMNC47883-20 GBMNC47884-20 GBMNC47885-20  Group[ 7 ] n: 10 ;id: IBIPP003-19 IBIPP035-20 IBIPP036-20 IBIPP053-20 IBIPP058-20 IBIPP085-20 IBIPP059-20 IBIPP104-20 IBIPP034-20 IBIPP084-20 |
| ***Leuctra prima* / *Leuctra* sp. ZB** |
| Partition 1  Score: 1  Proba: 3.877737e-03  nb groups:2 (1)  Group[ 1 ] n: 2 ;id: CROPL248-21 CROPL384-22  Group[ 2 ] n: 18 ;id: CROPL391-22 CROPL016-21 GST207-15 GST209-15 GST212-15 GST214-15 GST218-15 GST220-15 GST208-15 GST210-15 GST215-15 GST217-15 GST219-15 CROPL023-21 FBAQU1315-12 CROPL003-21 PLEAA205-20 PLEAA382-20  Partition 2  Score: 2  Proba: 1.317365e-01  nb groups:4 (3)  Group[ 1 ] n: 1 ;id: CROPL248-21  Group[ 2 ] n: 16 ;id: CROPL391-22 CROPL016-21 GST207-15 GST209-15 GST212-15 GST214-15 GST218-15 GST220-15 GST208-15 GST210-15 GST215-15 GST217-15 GST219-15 CROPL023-21 FBAQU1315-12 CROPL003-21  Group[ 3 ] n: 2 ;id: PLEAA205-20 PLEAA382-20  Group[ 4 ] n: 1 ;id: CROPL384-22  Partition 3  Score: 3  Proba: 3.632735e-01  nb groups:8 (7)  Group[ 1 ] n: 1 ;id: CROPL248-21  Group[ 2 ] n: 2 ;id: CROPL391-22 CROPL016-21  Group[ 3 ] n: 11 ;id: GST207-15 GST209-15 GST212-15 GST214-15 GST218-15 GST220-15 GST208-15 GST210-15 GST215-15 GST217-15 GST219-15  Group[ 4 ] n: 1 ;id: CROPL384-22  Group[ 5 ] n: 1 ;id: CROPL003-21  Group[ 6 ] n: 1 ;id: CROPL023-21  Group[ 7 ] n: 1 ;id: FBAQU1315-12  Group[ 8 ] n: 2 ;id: PLEAA205-20 PLEAA382-20 |
| **ASAP *p*-distance** |
| ***Brachyptera seticornis*** |
| Partition 1  Score: 1  Proba: 2.560241e-02  nb groups:3 (2)  Group[ 1 ] n: 6 ;id: BGMAY490-11 BGMAY502-11 CROPL049-21 BGMAY500-11 BGMAY501-11 CROPL046-21  Group[ 2 ] n: 48 ;id: CROPL055-21 FBAQU1304-12 GBEPT301-14 GBMND82052-21 GBMND82053-21 GBMND82054-21 GBMND82055-21 GBMND82056-21 CROPL392-22 GBMND82060-21 GBMND82061-21 GBMND82062-21 GBMND82065-21 GBMND82068-21 GBMND82070-21 GBMND82071-21 GBMND82072-21 GBMND82073-21 GBMND82074-21 GBMND82075-21 GBMND82076-21 GBMND82078-21 GBMND82081-21 GBMND82082-21 GBMND82083-21 GBMND82085-21 GBMND82086-21 GBMND82087-21 GBMND82089-21 GBMND82090-21 GBMND82091-21 GBMND82092-21 GBMND82094-21 PLEAA161-20 GBMND82057-21 GBMND82058-21 GBMND82059-21 GBMND82063-21 GBMND82067-21 GBMND82069-21 GBMND82077-21 GBMND82064-21 GBMND82080-21 GBMND82084-21 GBMND82088-21 GBMND82093-21 GBMND82066-21 GBMND82079-21  Group[ 3 ] n: 3 ;id: CROPL014-21 CROPL139-21 CROPL149-21  Partition 2  Score: 2  Proba: 5.528942e-01  nb groups:4 (3)  Group[ 1 ] n: 5 ;id: BGMAY490-11 BGMAY502-11 CROPL049-21 BGMAY500-11 BGMAY501-11  Group[ 2 ] n: 1 ;id: CROPL046-21  Group[ 3 ] n: 3 ;id: CROPL014-21 CROPL139-21 CROPL149-21  Group[ 4 ] n: 48 ;id: CROPL055-21 FBAQU1304-12 GBEPT301-14 GBMND82052-21 GBMND82053-21 GBMND82054-21 GBMND82055-21 GBMND82056-21 CROPL392-22 GBMND82060-21 GBMND82061-21 GBMND82062-21 GBMND82065-21 GBMND82068-21 GBMND82070-21 GBMND82071-21 GBMND82072-21 GBMND82073-21 GBMND82074-21 GBMND82075-21 GBMND82076-21 GBMND82078-21 GBMND82081-21 GBMND82082-21 GBMND82083-21 GBMND82085-21 GBMND82086-21 GBMND82087-21 GBMND82089-21 GBMND82090-21 GBMND82091-21 GBMND82092-21 GBMND82094-21 PLEAA161-20 GBMND82057-21 GBMND82058-21 GBMND82059-21 GBMND82063-21 GBMND82067-21 GBMND82069-21 GBMND82077-21 GBMND82064-21 GBMND82080-21 GBMND82084-21 GBMND82088-21 GBMND82093-21 GBMND82066-21 GBMND82079-21  Partition 3  Score: 3  Proba: 2.654691e-01  nb groups:5 (4)  Group[ 1 ] n: 5 ;id: BGMAY490-11 BGMAY502-11 CROPL049-21 BGMAY500-11 BGMAY501-11  Group[ 2 ] n: 3 ;id: CROPL014-21 CROPL139-21 CROPL149-21  Group[ 3 ] n: 1 ;id: CROPL046-21  Group[ 4 ] n: 47 ;id: CROPL055-21 FBAQU1304-12 GBEPT301-14 GBMND82052-21 GBMND82053-21 GBMND82054-21 GBMND82055-21 GBMND82056-21 CROPL392-22 GBMND82060-21 GBMND82061-21 GBMND82062-21 GBMND82065-21 GBMND82068-21 GBMND82070-21 GBMND82071-21 GBMND82072-21 GBMND82073-21 GBMND82074-21 GBMND82075-21 GBMND82076-21 GBMND82078-21 GBMND82081-21 GBMND82082-21 GBMND82083-21 GBMND82085-21 GBMND82086-21 GBMND82087-21 GBMND82089-21 GBMND82090-21 GBMND82091-21 GBMND82092-21 GBMND82094-21 PLEAA161-20 GBMND82057-21 GBMND82058-21 GBMND82059-21 GBMND82063-21 GBMND82067-21 GBMND82069-21 GBMND82077-21 GBMND82064-21 GBMND82080-21 GBMND82084-21 GBMND82088-21 GBMND82093-21 GBMND82066-21  Group[ 5 ] n: 1 ;id: GBMND82079-21 |
| ***Leuctra braueri*** |
| Partition 1  Score: 1  Proba: 3.717201e-02  nb groups:2 (1)  Group[ 1 ] n: 32 ;id: FBAQU194-09 FBAQU331-09 GBEPT1008-14 GBEPT2302-15 GBEPT2359-15 GBEPT2364-15 GBEPT2371-15 GBMIX1524-15 GBMIX1528-15 GBMIX1531-15 GBMIX2465-15 GBMIX2475-15 GBMIX2485-15 GBMNC47659-15 GBEPT2353-15 GBEPT2354-15 GBEPT2355-15 GBEPT2356-15 GBEPT2357-15 GBEPT2358-15  GBMIX1667-15 INTAP035-17 INTAP036-17 INTAP037-17 PLEAA096-20 PLEAA170-20 PLEAA413-20 GBMIX2463-15 GMGRI255-13 GBMNC47660-20 INTAP038-17 GST167-15  Group[ 2 ] n: 9 ;id: CROPL389-22 CROPL379-22 CROPL385-22 CROPL375-22 CROPL380-22 CROPL378-22 CROPL387-22 CROPL386-22 CROPL376-22  Partition 2  Score: 2  Proba: 6.606786e-01  nb groups:4 (2)  Group[ 1 ] n: 31 ;id: FBAQU194-09 FBAQU331-09 GBEPT1008-14 GBEPT2302-15 GBEPT2359-15 GBEPT2364-15 GBEPT2371-15 GBMIX1524-15 GBMIX1528-15 GBMIX1531-15 GBMIX2465-15 GBMIX2475-15 GBMIX2485-15 GBMNC47659-15 GBEPT2353-15 GBEPT2354-15 GBEPT2355-15 GBEPT2356-15 GBEPT2357-15 GBEPT2358-15 GBMIX1667-15 INTAP035-17 INTAP036-17 INTAP037-17 PLEAA096-20 PLEAA170-20 PLEAA413-20 GBMIX2463-15 GMGRI255-13 GBMNC47660-20 INTAP038-17  Group[ 2 ] n: 1 ;id: GST167-15  Group[ 3 ] n: 1 ;id: CROPL389-22  Group[ 4 ] n: 8 ;id: CROPL379-22 CROPL385-22 CROPL375-22 CROPL380-22 CROPL378-22 CROPL387-22 CROPL386-22 CROPL376-22  Partition 3  Score: 3  Proba: 6.487026e-01  nb groups:5 (4)  Group[ 1 ] n: 30 ;id: FBAQU194-09 FBAQU331-09 GBEPT1008-14 GBEPT2302-15 GBEPT2359-15 GBEPT2364-15 GBEPT2371-15 GBMIX1524-15 GBMIX1528-15 GBMIX1531-15 GBMIX2465-15 GBMIX2475-15 GBMIX2485-15 GBMNC47659-15 GBEPT2353-15 GBEPT2354-15 GBEPT2355-15 GBEPT2356-15 GBEPT2357-15 GBEPT2358-15 GBMIX1667-15 INTAP035-17 INTAP036-17 INTAP037-17 PLEAA096-20 PLEAA170-20 PLEAA413-20 GBMIX2463-15 GMGRI255-13 GBMNC47660-20  Group[ 2 ] n: 1 ;id: INTAP038-17  Group[ 3 ] n: 1 ;id: CROPL389-22  Group[ 4 ] n: 8 ;id: CROPL379-22 CROPL385-22 CROPL375-22 CROPL380-22 CROPL378-22 CROPL387-22 CROPL386-22 CROPL376-22  Group[ 5 ] n: 1 ;id: GST167-15 |
| ***Isoperla grammatica*** |
| Partition 1  Score: 1  Proba: 2.634731e-01  nb groups:6 (5)  Group[ 1 ] n: 7 ;id: CROPL154-21 CROPL265-21 CROPL338-21 CROPL276-21 CROPL342-21 CROPL292-21 CROPL205-21  Group[ 2 ] n: 4 ;id: CROPL318-21 GBMNC47877-20 FBAQU007-09 INTAP172-17  Group[ 3 ] n: 3 ;id: CROPL395-22 CROPL396-22 CROPL397-22  Group[ 4 ] n: 6 ;id: FIEPT075-11 PLEAA243-20 FIEPT076-11 FIEPT078-11 FIEPT079-11 PLEAA324-20  Group[ 5 ] n: 10 ;id: IBIPP003-19 IBIPP035-20 IBIPP036-20 IBIPP053-20 IBIPP058-20 IBIPP085-20 IBIPP059-20 IBIPP104-20 IBIPP034-20 IBIPP084-20  Group[ 6 ] n: 15 ;id: GBMNC47878-20 GBMNC47879-20 GBMNC47880-20 GBMNC47881-20 GBMNC47886-20 GBMNC47887-20 GBMNC47888-20 GBMNC47889-20 GBMNC47882-20 GBMNC47883-20 GBMNC47890-20 GBMNC47891-20 GBMNC47892-20 GBMNC47884-20 GBMNC47885-20  Partition 2  Score: 2  Proba: 6.966068e-01  nb groups:3 (2)  Group[ 1 ] n: 27 ;id: CROPL154-21 CROPL265-21 CROPL338-21 CROPL276-21 CROPL342-21 CROPL292-21 CROPL205-21 CROPL318-21 GBMNC47877-20 FBAQU007-09 INTAP172-17 FIEPT075-11 PLEAA243-20 FIEPT076-11 FIEPT078-11 FIEPT079-11 PLEAA324-20 IBIPP003-19 IBIPP035-20 IBIPP036-20 IBIPP053-20 IBIPP058-20 IBIPP085-20 IBIPP059-20 IBIPP104-20 IBIPP034-20 IBIPP084-20  Group[ 2 ] n: 3 ;id: CROPL395-22 CROPL396-22 CROPL397-22  Group[ 3 ] n: 15 ;id: GBMNC47878-20 GBMNC47879-20 GBMNC47880-20 GBMNC47881-20 GBMNC47886-20 GBMNC47887-20 GBMNC47888-20 GBMNC47889-20 GBMNC47882-20 GBMNC47883-20 GBMNC47890-20 GBMNC47891-20 GBMNC47892-20 GBMNC47884-20 GBMNC47885-20  Partition 3  Score: 3  Proba: 7.325349e-01  nb groups:7 (6)  Group[ 1 ] n: 7 ;id: CROPL154-21 CROPL265-21 CROPL338-21 CROPL276-21 CROPL342-21 CROPL292-21 CROPL205-21  Group[ 2 ] n: 2 ;id: CROPL318-21 GBMNC47877-20  Group[ 3 ] n: 2 ;id: FBAQU007-09 INTAP172-17  Group[ 4 ] n: 3 ;id: CROPL395-22 CROPL396-22 CROPL397-22  Group[ 5 ] n: 6 ;id: FIEPT075-11 PLEAA243-20 FIEPT076-11 FIEPT078-11 FIEPT079-11 PLEAA324-20  Group[ 6 ] n: 15 ;id: GBMNC47878-20 GBMNC47879-20 GBMNC47880-20 GBMNC47881-20 GBMNC47886-20 GBMNC47887-20 GBMNC47888-20 GBMNC47889-20 GBMNC47882-20 GBMNC47883-20 GBMNC47890-20 GBMNC47891-20 GBMNC47892-20 GBMNC47884-20 GBMNC47885-20  Group[ 7 ] n: 10 ;id: IBIPP003-19 IBIPP035-20 IBIPP036-20 IBIPP053-20 IBIPP058-20 IBIPP085-20 IBIPP059-20 IBIPP104-20 IBIPP034-20 IBIPP084-20 |
| ***Leuctra prima / Leuctra* sp. ZB** |
| Partition 1  Score: 1  Proba: 7.332854e-03  nb groups:2 (1)  Group[ 1 ] n: 2 ;id: CROPL248-21 CROPL384-22  Group[ 2 ] n: 18 ;id: CROPL391-22 CROPL016-21 GST207-15 GST209-15 GST212-15 GST214-15 GST218-15 GST220-15 GST208-15 GST210-15 GST215-15 GST217-15 GST219-15 CROPL023-21 FBAQU1315-12 CROPL003-21 PLEAA205-20 PLEAA382-20  Partition 2  Score: 2  Proba: 1.357285e-01  nb groups:4 (3)  Group[ 1 ] n: 1 ;id: CROPL248-21  Group[ 2 ] n: 16 ;id: CROPL391-22 CROPL016-21 GST207-15 GST209-15 GST212-15 GST214-15 GST218-15 GST220-15 GST208-15 GST210-15 GST215-15 GST217-15 GST219-15 CROPL023-21 FBAQU1315-12 CROPL003-21  Group[ 3 ] n: 2 ;id: PLEAA205-20 PLEAA382-20  Group[ 4 ] n: 1 ;id: CROPL384-22  Partition 3  Score: 3  Proba: 3.413174e-01  nb groups:8 (7)  Group[ 1 ] n: 1 ;id: CROPL248-21  Group[ 2 ] n: 2 ;id: CROPL391-22 CROPL016-21  Group[ 3 ] n: 11 ;id: GST207-15 GST209-15 GST212-15 GST214-15 GST218-15 GST220-15 GST208-15 GST210-15 GST215-15 GST217-15 GST219-15  Group[ 4 ] n: 1 ;id: CROPL384-22  Group[ 5 ] n: 1 ;id: CROPL003-21  Group[ 6 ] n: 1 ;id: CROPL023-21  Group[ 7 ] n: 1 ;id: FBAQU1315-12  Group[ 8 ] n: 2 ;id: PLEAA205-20 PLEAA382-20 |

**Supplementary Table S5.** Ranges of intraspecific uncorrected pairwise distances (*p*-distances) within and between groups (G1–G3) of the species *Brachyptera seticornis*, as indicated on the phylogenetic tree and network (see Figures 2.A and 2.B). The maximum genetic distance within groups is highlighted in yellow, while the range of genetic distance between groups is shown in white.

|  | **G1** | **G2** | **G3** |
| --- | --- | --- | --- |
| **G1** | 1.06 % |  |  |
| **G2** | 1.37 % - 2.28 % | 0.15 % |  |
| **G3** | 1.37 % - 2.28 % | 1.37 % - 2.13 % | 0.91 % |

**Supplementary Table S6.** Ranges of intraspecific uncorrected pairwise distances (*p*-distances) within and between groups (G1–G2) of the species *Leuctra braueri*, as indicated on the phylogenetic tree and network (see Figures 3.A and 3.B). The maximum genetic distance within groups is highlighted in yellow, while the range of genetic distance between groups is shown in white.

|  | **G1** | **G2** |
| --- | --- | --- |
| **G1** | 0.61 % |  |
| **G2** | 1.84 % - 2.46 % | 0.61 % |

**Supplementary Table S7.** Ranges of intraspecific uncorrected pairwise distances (*p*-distances) within and between groups (G1–G4) of the species *Leuctra prima* and *Leuctra* sp. ZB, as indicated on the phylogenetic tree and network (see Figures 4.A and 4.B). The maximum genetic distance within groups is highlighted in yellow, while the range of genetic distance between groups is shown in white.

|  | **G1** | **G2** | **G3** | **G4** |
| --- | --- | --- | --- | --- |
| **G1** | 0 % |  |  |  |
| **G2** | 3.65 % | 0 % |  |  |
| **G3** | 12.79 % - 13.55 % | 12.02 % - 12.94 % | 1.37 % |  |
| **G4** | 12.18 % - 12.32 % | 12.79 % - 12.94 % | 3.20 % - 3.65 % | 0.30 % |

**Supplementary Table S8.** Ranges of intraspecific uncorrected pairwise distances (*p*-distances) within and between groups (G1–G6) of the species *Isoperla grammatica*, as indicated on the phylogenetic tree and network (see Figures 5.A and 5.B). The maximum genetic distance within groups is highlighted in yellow, while the range of genetic distance between groups is shown in white.

|  | **G1** | **G2** | **G3** | **G4** | **G5** | **G6** |
| --- | --- | --- | --- | --- | --- | --- |
| **G1** | 1.09 % |  |  |  |  |  |
| **G2** | 9.64 % - 10.4 % | 0.16 % |  |  |  |  |
| **G3** | 10.1 % - 11.0 % | 8.55 % - 9.02 % | 0.47 % |  |  |  |
| **G4** | 9.33 % - 10.4 % | 7.93 % - 8.55 % | 1.55 % - 2.78 % | 1.71 % |  |  |
| **G5** | 9.80 % - 11.0 % | 8.71 % - 9. 49 % | 3.89 % - 4.67 % | 2.49 % - 4.20 % | 1.71 % |  |
| **G6** | 9.95 % - 11.7 % | 9.02 % - 10.3 % | 4.51 % - 5.61 % | 2.49 % - 4.51 % | 2.33 % - 3.42 % | 1.71 % |
